# Partial loss of interleukin 2 receptor gamma function in pigs provides mechanistic insights for the study of human immunodeficiency syndrome

**DOI:** 10.18632/oncotarget.10812

**Published:** 2016-07-24

**Authors:** Yun-Jung Choi, Kiho Lee, Woo-Jin Park, Deug-Nam Kwon, Chankyu Park, Jeong Tae Do, Hyuk Song, Seong-Keun Cho, Kwang-Wook Park, Alana N. Brown, Melissa S. Samuel, Clifton N. Murphy, Randall S. Prather, Jin-Hoi Kim

**Affiliations:** ^1^ Animal Biotechnology to Stem Cell and Regenerative Biotechnology, Humanized Pig Research Center (SRC), Konkuk University, Seoul, Republic of Korea; ^2^ Department of Animal and Poultry Sciences, Virginia Tech, Blacksburg, VA, USA; ^3^ Division of Animal Science, National Swine Resource and Research Center, University of Missouri, Columbia, MO, USA; ^4^ National Swine Resource and Research Center, University of Missouri, Columbia, MO, USA; ^5^ Department of Animal Science, Pusan National University, Miryang, Gyeongnam, Republic of Korea; ^6^ Department of Animal Science and Technology, Sunchon National University, Suncheon, Jeonnam, Republic of Korea

**Keywords:** IL2RG, X-SCID, immunodeficiency, somatic cell nuclear transfer, TALEN, Pathology Section

## Abstract

In this study, we described the phenotype of monoallelic interleukin 2 receptor gamma knockout (*mIL2RG*^+/Δ69-368^ KO) pigs. Approximately 80% of *mIL2RG*^+/Δ69-368^ KO pigs (8/10) were athymic, whereas 20% (2/10) presented a rudimentary thymus. The body weight of *IL2RG*^+/Δ69-368^KO pigs developed normally. Immunological analysis showed that *mIL2RG*^+/Δ69-368^ KO pigs possessed CD25^+^CD44- or CD25-CD44^+^ cells, whereas single (CD4 or CD8) or double (CD4/8) positive cells were lacking in *mIL2RG*^+/Δ69-368^ KO pigs. CD3^+^ cells in the thymus of *mIL2RG*^+/Δ69-368^ KO pigs contained mainly CD44^+^ cells and/or CD25^+^ cells, which included FOXP3^+^ cells. These observations demonstrated that T cells from *mIL2RG*^+/Δ69-368^ KO pigs were able to develop to the DN3 stage, but failed to transition toward the DN4 stage. Whole-transcriptome analysis of thymus and spleen, and subsequent pathway analysis revealed that a subset of genes differentially expressed following the loss of IL2RG might be responsible for both impaired T-cell receptor and cytokine-mediated signalling. However, comparative analysis of two *mIL2RG*^+/Δ69-368^ KO pigs revealed little variability in the down- and up-regulated gene sets. In conclusion, *mIL2RG*^+/Δ69-368^ KO pigs presented a T-B+NK- SCID phenotype, suggesting that pigs can be used as a valuable and suitable biomedical model for human SCID research.

## INTRODUCTION

Severe combined immunodeficiency (SCID) occurs in various species, including humans [1], at a frequency as high as one per 50,000 live births [2]. Generally, SCID can be classified according to the cause of the immunodeficiency, and it includes impaired cytokine-mediated signalling, defective V(D)J recombination, impaired pre-T-cell receptor signalling, and metabolic enzyme deficiencies [1–3]. Although mice with disrupted SCID-causing genes have provided important insights into the human disease, not all the SCID mice have phenotypes that resemble those in human SCID patients. In humans, most SCID patients are reported to have impaired cytokine-mediated signalling in immune cells [4]. Common gamma chain (γC), which is encoded by a gene located on the X chromosome, is essential for normal lymphocyte development as a functional interleukin-2 receptor subunit (IL2RG) [5]. Janus kinase 3 (JAK3) is required for signal transduction by γC-containing receptors [6]. Therefore, the phenotypes and characteristics of X-SCID and JAK3-SCID mice are identical or highly similar. However, one difference is that most of X-SCID affects male mice, whereas JAK3-SCID affects both male and female mice.

Many researchers have used pigs to develop surgical and implant technologies, and in the study of regenerative medicine, skin grafts, microbial disease, and a host of other applications, for which rodents are inadequate [7]. However, their genetic modification remains challenging owing to the difficulty in generating knock-out (KO) pigs and the lack of germline transferable embryonic stem cells [8–10]. Recently, important tools have been developed for use in gene knockout studies. For example, designed mega-nucleases can induce double-stranded breaks (DSBs) at specific sites in the genome, resulting in random modifications through non-homologous end joining (NHEJ) [11–13]. Recent achievements in the use of mega-nucleases such as Zinc-finger nucleases (ZFNs) [11, 12], transcription activator-like effector nucleases (TALENs) [14–16], and CRISPR/Cas9 [17] have also suggested that KO pigs can be efficiently generated. Recently, we and other groups have described X-linked or autosomal SCID-like pigs that were created by disrupting interleukin 2 receptor gamma (IL2RG) [18, 19], autosomal Rag-1/2 [20], and RAG-2 SCID [21] or by natural breeding [22]. These SCID pigs showed several defects in the architecture and composition of secondary lymphoid organs.

Despite some phenotypic characterisations and functional studies being performed in SCID animals, little is known about the molecular basis of the different phenotypes of SCID in pig. In the present study, we generated monoallelic IL2RG (*mIL2RG*^+/Δ69-368^) KO pigs and investigated patterns of gene expression during their immune development in order to further explore our understanding of immune responses in X-linked SCID.

## RESULTS

### Production of *mIL2RG*^+/Δ69-368^ KO pigs by utilizing TALENs

**Figure 1:**
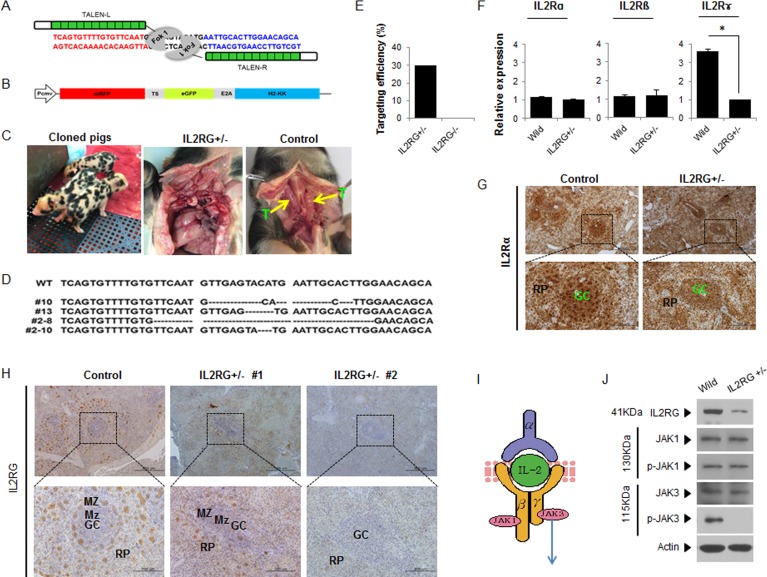
Production of *mIL2RG*^+/Δ69-368^ KO pigs using TALENs **A.** TALENs designed to cause mutations in Exon 2 of IL*2RG*. **B.** Donor vector for analysis of targeting efficacy. **C.** Underdeveloped thymus in *mIL2RG*^+/Δ69-368^ KO pig. Thymus was underdeveloped or absent in *mIL2RG*^+/Δ69-368^ KO pigs. T indicates thymus. **D.** Targeted mutation of *IL2RG* using TALENs. Deleted bases were indicated by dashes. **E.** Targeting efficacy. **F.** RT-qPCR analysis. **G.** and **H.** Localization of IL2Rα (G) and γ (H) protein expression in the spleen of control (WT) and *mIL2RG*^+/Δ69-368^ KO pigs. **I.** Cartoon showing a **representative** diagram of IL2 signaling. **J.** Western blot analysis with IL2RG, JAK1, p-JAK1, JAK3, and p-JAK3 antibodies.

TALEN was designed to cause DSBs on exon 2 of *IL2RG* (Figure 1A). A reporter construct containing TALEN recognition sites was used to identify cells expressing TALEN sets (Figure 1B and Supplementary Figure 1). The efficiency of gene targeting was 30% (9/30) (Figure 1E). The sequencing results revealed the presence of insertions or deletions (indels) from the TALENs in *IL2RG*. Mutation patterns varied remarkably among the positive colonies. Four *IL2RG*^+/−^ cell colonies containing deletions from 4 to 29bp in size (Figure 1D) were selected as donors for somatic cell nuclear transfer (SCNT). Through SCNT, 11 *IL2RG* KO pigs (*mIL2RG*^+/Δ69-368^) were produced (Supplementary Table 1); however, nine died in the first week post-birth. At 3-months-old, one pig was subjected to further analysis. At necropsy, only two of 10 *mIL2RG*^+/Δ69-368^ KO pigs had a rudimentary thymus (Figure 1C).

To examine any off-target effects in *mIL2RG*^+/Δ69-368^ KO pigs, we screened the pig genome and predicted nine off-target sites for the *IL2RG* gene. As shown in Supplementary Figure 2B, we could not find any off-target sites in the *mIL2RG*^+/Δ69-368^ KO pigs derived from our TALEN targeting system. According to our observation, *mIL2RG*^+/Δ69-368^ KO pigs showed more anxiety and hyperactive behaviour, such as fidgeting and impulsive actions, compared with age-matched wild type pigs (WT).

**Figure 2:**
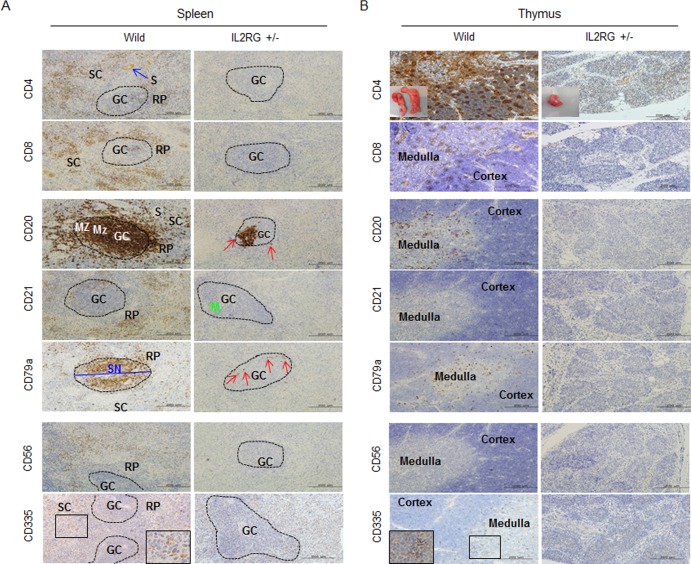
Analysis of T-, B-, and NK-specific biomarker expression patterns in spleen (A) and thymus (B) T cells (CD4 and CD8), B cells (CD20, CD21, and CD79a), NK cells (CD56 and CD 335) positive signal in WT-derived spleen tissues were strongly detected, whereas a few CD20 and CD79a positive cells in *mIL2RG*^+/Δ69-368^ KO pigs, which is a critical factors for B cell development, were detected. However, biomarkers expression of T and NK cells in thymus of *mIL2RG*^+/Δ69-368^ KO pigs were not detected. GC, germinal center; RP, red pulp; S, splenic sinusoids; M, mantle; SC, splenic cords; SN, splenic nodule.

### Analysis of IL2RG neighbouring gene expression

As shown in Figure 1F, the abundance of *IL2RG* mRNA was dramatically reduced in the *mIL2RG*^+/Δ69-368^KO pigs compared to WT; while there is no difference that was observed in the levels of *IL2RA* and *IL2RB* mRNA expression. When protein expression of IL2RG in the spleen was determined using immunohistochemical analysis, IL2RG positive cell numbers in *mIL2RG*^+/Δ69-368^KO pig were significantly lower or undetectable compared with WT (Figure 1H), whereas IL2RA-positive signals were not changed (Figure 1G). Since two *mIL2RG*^+/Δ69-368^ KO pigs have small thymus, the role of the γ subunit in IL2R-mediated signalling from two *mIL2RG*^+/Δ69-368^ pig-derived thymus was examined. As shown in Figure 1I and 1J, the level of JAK3 protein, a downstream target of IL2RG, was not changed in the thymus of *mIL2RG*^+/Δ69-368^ KO pig compared to the WT. Also, the level of JAK1 and pJAK1 protein in *mIL2RG*^+/Δ69-368^ KO pigs was not altered. Even though JAK3 protein level in the *mIL2RG*^+/Δ69-368^ KO pig was not changed or slightly decreased, pJAK3 level was significantly decreased and undetectable compared with WT. Our observations suggest a role of a γC and JAK3 pathway in X-SCID pigs.

### *mIL2RG*^+/Δ69-368^ KO pigs are T- and NK-cell negative, but B cell positive

In this study, two out of ten *mIL2RG*^+/Δ69-368^ KO pigs had a small, but detectable, thymus (Figure 1C). As shown in Figure 2A, *mIL2RG*^+/Δ69-368^ KO pigs resembled the T^−^B^+^NK^−^ SCID human phenotype, although the level of B cells was remarkably reduced. In WT, CD20 and CD79a positive cells were abundant in the red pulp (RP) and splenic nodule (SN), whereas very small populations of CD20 and CD79a positive cells were observed in *mIL2RG*^+/Δ69-368^ KO pigs in the marginal zone (MZ) of the germinal centre (GC). In the thymus of *mIL2RG*^+/Δ69-368^ KO piglets, the thymic medulla and cortex were significantly atrophied (Figure 2B). As expected, IL2RG KO pigs lacked single positive (SP) cells (CD4^+^ or CD8^+^ T cells) and double positive (DP) cells (CD4^+^CD8^+^ T cells). mIL2RG^+/Δ69-368^ KO pigs also lacked CD56 and CD335 positive cells, indicating the absence of NK cells.

### CD3 expression is detected in thymic and splenic double-negative (CD4^−^CD8^−^) cells in WT and *mIL2RG*^+/Δ69-368^ KO pigs

**Figure 3:**
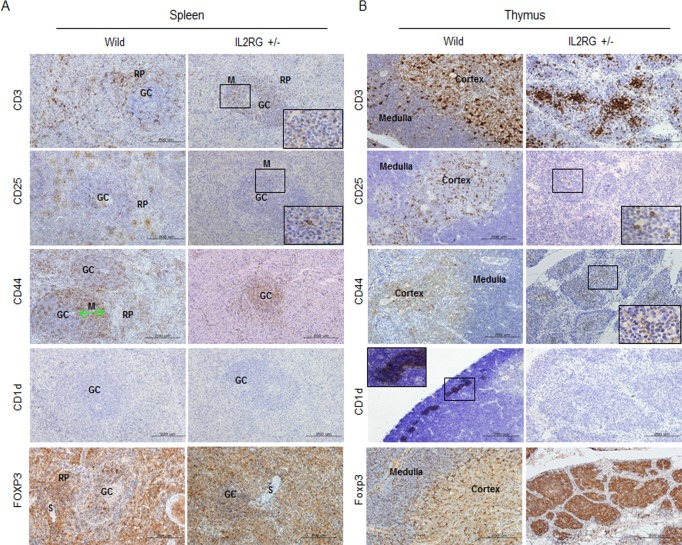
Expression of T-cell subsets markers in spleen and thymus of *mIL2RG*^+/Δ69-368^ KO In the spleen **A.** and thymus **B.** of *IL2RG*^+/Δ69-368^ KO pigs, expression of CD3, CD25, and CD44 CD1d were rarely found compared with WT spleen and thymus. CD1d did not show any expression in spleen and thymus of *IL2RG*^+/Δ69-368^ KO. GC, germinal center; RP, red pulp; S, splenic sinusoids; M, mantle; SC, splenic cords; SN, splenic nodule.

Strong signals for CD3 positive T cells in the RP and MZ of the GC were detected in the spleen of WT pigs. However, only moderately positive cells were detected in the MZ within GC of *mIL2RG*^+/Δ69-368^ KO pigs (Figure 3A). High numbers of CD25- and CD44-positive cells were also detected in RP and SN, whereas these cells were only detected in the GC of mIL2RG^+/Δ69-368^ KO pigs. Furthermore, CD1d-positive cells were absent in the spleen from both WT and *mIL2RG*^+/Δ69-368^ KO pigs, however, distinct CD1d-positive cells were only detected in the thymus from WT pigs (Figure 3A and 3B). Although CD25-, CD44-, and CD3-positive cells in the thymus of WT and *mIL2RG*^+/Δ69-368^ KO pigs were found mainly in the medulla area, their expression was decreased in the *mIL2RG*^+/Δ69-368^ KO pigs compared to WT (Figure 3B). In addition, FOXP3-positive cells in both spleen and thymus of WT showed similar staining patterns, compared to those in *mIL2RG*^+/Δ69-368^ KO pigs. Macrophage infiltration was detected in the livers of all *mIL2RG*^+/Δ69-368^ KO pigs examined (Supplementary Figure 4).

**Figure 4:**
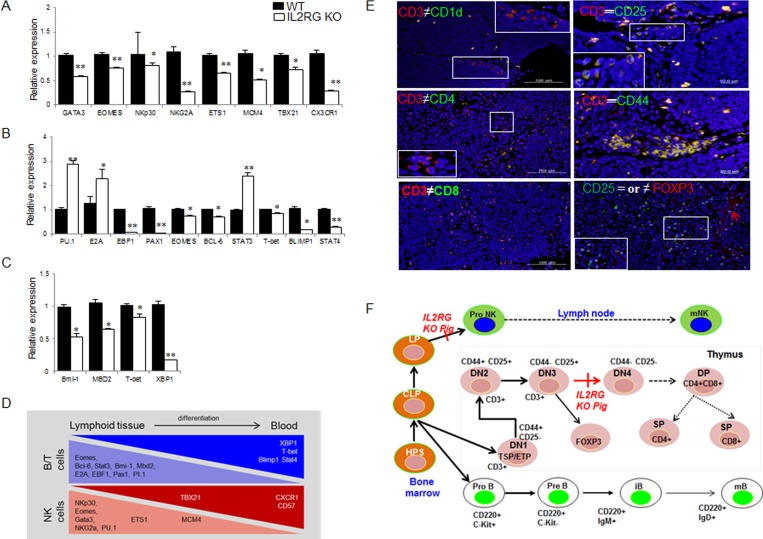
Comparison of B-, T-, and NK-cell specific gene expression by using RT-qPCR and immunofluorescence analysis in WT and *mIL2RG*^+/Δ69-368^ KO pig mRNA expression of NK **A.**, **B.**, and T **C.** cells development related genes in *mIL2RG*^+/Δ69-368^ KO pig-derived spleen, thymus, and lymph node were significantly down-regulated, whereas expression of PU.1, E2A, and STAT3 encoding early B cell development related genes were up-regulated. **D.** Cartoon showing major events of T/B/NK cell development. Developing thymocytes, which enter into thymus and migrate into blood, show differential gene expression profiles that modulate the discrete stages of the T/B/NK-cell development. **E.** To identify T cell developmental stages in *mIL2RG*^+/Δ69-368^ KO pigs-derived thymus, CD3 positive cells were co-localized with CD1d or CD4, CD8, CD25, and CD 44 antibodies. Also, CD25 positive cells are colocalized with FOXP3 antibody. **F.** T cell developmental blocks largely rely on data from targeted mutation studies of mIL2RG^+/Δ69-368^ KO pigs.

Using RT-qPCR, we found that various genes involved in T-, B-, and NK-cell maturation or differentiation are dysregulated in the *mIL2RG*^+/Δ69-368^ KO pigs (Figure 4A-4D). Specifically, the expression of several genes related to lymphocyte development was lower in the spleen and thymus of *mIL2RG*^+/Δ69-368^ KO pigs than in the WT. Interestingly, expression of genes that are important in early B cell development, such as *PU-1*, *E2A*, and *STAT3*, was up-regulated in the spleen of *mIL2RG*^+/Δ69-368^ KO pigs compared to WT, whereas most of the genes expressed during late B-cell development were significantly down-regulated.

To identify subtypes of cells responsible for the CD3 positive T cells in the spleen and thymus, CD3 expression was co-localized with CD25 or CD44, CD1d, and FOXP3. As shown in Figure 4E and Supplementary Figures 3.1-3.4, most of the CD3-positive cells in *mIL2RG*^+/Δ69-368^ KO pigs were also positive for CD44 (CD3^+^CD44^+^) completely and/or partially with CD+25 positive cells (CD3^+^CD25^+^). However, no CD4 or CD8 positive signals were detected (Figure 4E). Furthermore, some of the CD25-positive cells were partially co-localized with FOXP3 expression. Taken together, these observations suggest that T-cell development in *IL2RG*^+/Δ69-368^ KO pigs is arrested between the DN3 (CD44^−^CD25^+^) and DN4 stages (CD44^−^CD25^−^) due to an impaired transition (Figure 4F), indicating that this is the first critical difference between the pig and mouse immune system.

### Impact of partial IL2RG deficiency on genome-wide gene expression patterns in the spleen and thymus

**Figure 5:**
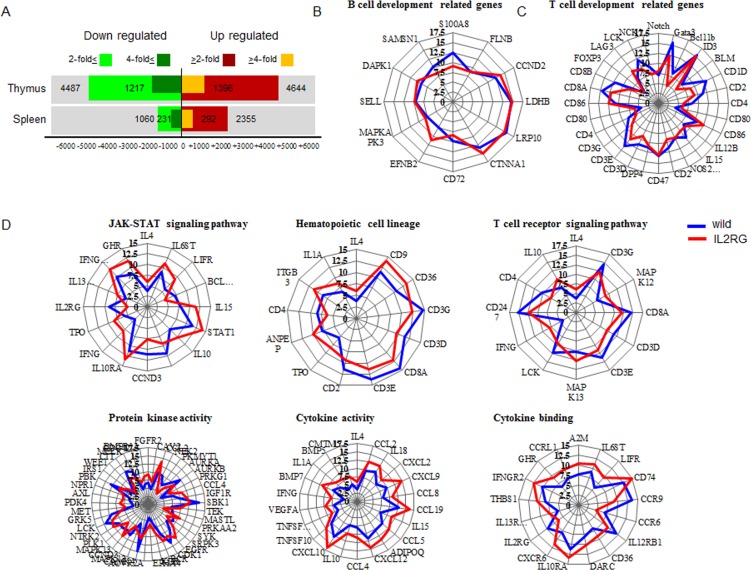
Microarray and ingenuity pathway analysis **A.** Up- and down- regulated gene numbers in mIL2RG^+/Δ69-368^ KO pig-derived spleen and thymus compared with WT. IPA indicate that B cell development related genes in spleen showed similar expression pattern compared to WT **B.**, whereas T cell development related genes showed slightly down-regulated expressions in *mIL2RG*^+/Δ69-368^ KO pigs-derived thymus than those of WT **C.** To predict T cell development-related gene expression profiles in in mIL2RG^+/Δ69-368^ KO pig-derived thymus, each T-cell signaling pathways were analyzed using IPA **D.** JAK-STAT signaling pathway including hematopoietic cell lineage, T cell receptor signaling pathway, and protein kinase activity in *mIL2RG*^+/Δ69-368^ KO pig derived-thymus showed very similar expression patterns compared to those of WT. Of note, cytokine activity and cytokine binding-related gene expression in *mIL2RG*^+/Δ69-368^ KO pig-derived thymus are significantly upregulated than those of WT. Blue and red color line indicate WT and *mIL2RG*^+/Δ69-368^ KO pigs, respectively.

Eight of 10 *mIL2RG*^+/Δ69-368^ KO pigs lacked thymus. Therefore, global gene expression patterns in thymus and spleen were analysed from the two *IL2RG* KO pigs with thymus using a porcine (V2) gene expression microarray composed of 43,803 probe sets (Supplementary Figure 5); their expression patterns were compared to WT. After unpaired *t*-test analyses were performed with Bonferroni corrections, 3405 and 523 (9131 and 2613) genes with a ≥2.0- and 4.0-fold change expression in spleen- (thymus-) derived genes (*p* < 0.05), were identified. Among these, 2355 and 292 (3644 and 1396) genes in spleen (thymus) were up-regulated, whereas 1050 and 231 (4487 and 1217) genes were down-regulated in *mIL2RG*^+/Δ69-368^ KO pigs compared with WT (Figure 5A). Comparison between the two *mIL2RG*^+/Δ69-368^ KO pigs showed minor variability in the down- and up-regulated gene sets.

To better understand their biological roles, genes that were significantly dysregulated in the spleen and thymus of *mIL2RG*^+/Δ69-368^ KO pigs were used to establish gene ontology (GO) classification categories using the DAVID tool. There are three ontologies in GO: cellular component, molecular function, and biological process [23]. In the GO classification of thymus, the top 10 enriched biological processes in thymus from the GO analysis were categorised according to their functional role, as shown in Supplementary Figure 6 and Tables 6–9. These biological groups covered inflammatory response and immune response. In the spleen, 10 enriched biological processes from the GO analysis were categorised according to their functional roles, as shown in Supplementary Figure 8 and Tables 10-13.

The quantization radar chart in Figure 5B-5D shows the change in each gene expressions associated with T-, B-, and NK-cell lineages in the mIL2RG^+/Δ69-368^ KO pigs in comparison to the baseline of the WT. The map shows the expression difference in mIL2RG^+/Δ69-368^ KO pigs and WT. The expression profile of genes related to B-cell development in the *mIL2RG*^+/Δ69-368^ KO pigs followed a similar pattern to that of WT. Although the expressions of *IL2RG, CCND3, IL12RB1*, and *BCL2L1*, which are involved in the JAK-STAT signalling pathway, and/or CD3, CD4, CD8, and CD1d, which are involved in the development of T cells, are significantly decreased in *mIL2RG*^+/Δ69-368^ KO pigs, the expressions of *IFNG, IFNGR2, IL10RA, IL13RA1, TPO, GHR, IL4, IL6ST, LIFR, IL10, IL15*, and are *STAT1* mRNAs significantly increased (Figure 5C). The decreased expression of these genes was consistent with findings from previous studies [19], although the role of increased marker gene expression in *mIL2RG*^+/Δ69-368^ KO pigs remains unclear. Of note, the expression of genes associated with cytokine activity and binding pathways in *mIL2RG*^+/Δ69-368^ KO pigs is increased compared to WT (Figure 5D). The expression patterns of genes involved in the haematopoietic cell linage pathway, T-cell receptor signalling pathway, and protein kinase signalling pathway in *mIL2RG*^+/Δ69-368^ KO pigs were the same as those found for WT.

### Putative IL2RG interaction network analysis

**Figure 6:**
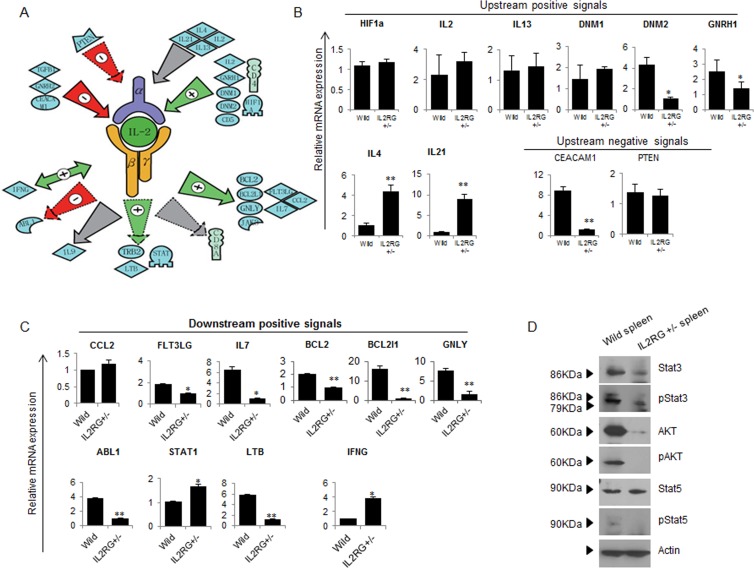
Studio pathway analysis of IL2RG signaling in *mIL2RG*^+/Δ69-368^ KO pig **A.** Functional correlation and interaction of IL2RG signaling using the Pathway Studio software (Ariadne Genomics Inc.; Rockville, MD, USA). All genes are shown by their gene symbols. Direct and indirect regulation are indicated by colored lines and dotted gray lines, respectively. **B.** and **C.** RT-qPCR validation of IL2RG upstream (B)- and downstream (C)-related gene expression proposed by Studio Pathway Analysis. **D.** A western blot analysis of AKT, pAKT, STAT5, pSTAT5, STAT3 and p STAT3 was performed in thymus of WT and mIL2RG^+/Δ69-368^ KO pigs. B actin mRNA expression, which used as an internal positive control in both of WT and mIL2RG^+/Δ69-368^ KO pig-derived thymus, was found to be similar. The data are representative of three experiments.

In this study, we examined a novel computational workflow to design a therapeutic strategy using the Pathway Studio Analysis (PSA). Using sub-network enrichment analysis (Figure 6A), we identified six proteins located downstream of IL2RG-derived thymus. Among them, we identified 10 positive signals, one negative signal, and one mutual signal, respectively. Likewise, we identified four signals located upstream of IL2RG. In spleen, we identified four negative signals on *IL2RG* gene expression, and seven positive signals (Figure 6B). Next, we examined whether the putative interaction networks are functional. As shown in Figure 6B and 6C, the data sets obtained from a representative potential signalling pathway were reconfirmed by RT-qPCR analysis. Among the upstream positive signals, *DNM2* and *GNRH1* gene expression in *IL2RG*^+/Δ69-368^ pigs were lower, whereas the expression of *IL-4* and *IL-21* mRNAs was significantly higher. However, most factors in *mIL2RG*^+/Δ69-368^ KO pigs were not altered compared to those in WT (Figure 6B). Of the negative upstream signals, CEACAM1 gene expression was significantly lower, whereas the level of *PTEN* was unchanged. Unlike the upstream signalling pathway, most of the downstream signal-related factors accurately reflect the predicted putative IL2RG interaction network (Figure 6C).

The mRNAs expression level of *STAT1* and *STAT3*, which are involved in NK cell development, growth, and activation, was significantly higher in *mIL2RG*^+/Δ69-368^ KO pigs (Figure 6C). However, *EOMES*, *T-bet*, and *STAT-4* mRNAs expression was significantly down-regulated (Figure 4). As shown in Figure 6D, total STAT3, p-STAT3, p-STAT5, total AKT, and p-AKT protein expressions were significantly decreased in the *IL2RG*^+/Δ69-368^ KO pigs compared to WT. Therefore, these results support the notion that the γC -JAK3-STAT5 pathway in *mIL2RG*^+/Δ69-368^ KO pigs is inactive during T-, B-, and NK-cell maturation/differentiation.

To obtain further insights into the cellular pathways affected by differentially expressed genes in IL2RG KO thymus and spleen, a network analysis using Ingenuity Pathway Analysis (IPA) was performed. Each case was linked to at least 24 different cellular pathways, even if a small amount of cross-linking was observed (Supplementary Figure 7.1). From the thymus data, network 4 showed connective tissue disorders, immunological disease, and inflammatory disease, included 26 genes, and showed a network with *IFNG* as a central node (Supplementary Figure 7.2). Up-regulated genes, such as *IFNG*, and down-regulated genes, such as *CD4*, *CD2*, and *RGN*, were found to play key roles in network 4. Network-5 showed a network with *IL4*, *IL10*, and *IL12* complexes as a central node (Supplementary Figure 7.3). Network 9 presented cell cycle, DNA replication, recombination and repair, cell death, and survival, and included 25 genes, which contain a network of *NF-kB* complexes, *SRC* families, and *CD9* as central nodes (Supplementary Figure 7.4). Network 14 and 15 show networks with *AKT*, and *APP*, *BCL2L1*, *IGM* as a central node (Supplementary Figure 7.5 and 7.6), which show cancer, haematological disease, and immunological disease, respectively.

*IFN-γ* mRNA expression in the *mIL2RG*^+/Δ69-368^ KO pigs was significantly up-regulated (Figure 6C), whereas *STAT4* mRNA expression (Figure 5D), which is required for IL12-induced IFN-γ production [24], was significantly down-regulated. These seemingly inconsistent results provide further information for a more detailed evaluation of the functional loss of *IL2RG* gene. IPA from thymus showed that differentially expressed genes in *IL2RG*^+/Δ69-368^ KO pigs were involved in the following processes: proliferation of immune cells (Supplementary Figure 10.1), quantity of T lymphocytes (Supplementary Figure 10.2), organ inflammation (Supplementary Figure 10.3), and macrophage infiltration (Supplementary Figure 10.4). Of note, key genes involved in the proliferation of immune cells and quantity of T lymphocytes were down-regulated, whereas the expression of genes involved in organ inflammation and infiltration were up-regulated. As shown in Supplementary Figures 4 and 7.2, higher *IFN-γ* mRNA expression in *IL2RG*^+/Δ69-368^ KO pigs may be caused by high levels of macrophage infiltration. Taken together, these observations suggest why *mIL2RG*^+/Δ69-368^ KO pigs exhibited increased *INF-γ* mRNA expression. Finally, IPA from the spleen data showed several networks. Among them, network 2 shows infectious disease, cell morphology, cell-to-cell signaling, and interaction, and include 19 genes (Supplementary Figure 9).

## DISCUSSION

Previous studies demonstrated that X-SCID phenotype with T^−^B^+^NK^−^ in humans [25] and a T^−^B^−^NK^−^ in mice [26] was caused by mutations of *IL2RG* gene. Here, we found that monoallelic disruption of pig *IL2RG* gene showed very closely similar characteristics to human SCID with T^−^B^+^NK^−^ phenotype rather than that of *IL2rg* KO mouse. This observation strongly suggests that *mIL2RG*^+/Δ69-368^ KO pigs can cover the significant differences between mouse and man. Taken together, our observations suggested that monoallelic *IL2RG* KO pigs may provide more mechanistic insights for the study of human immunodeficiency syndrome.

In immunohistological analysis, *mIL2RG*^+/Δ69-368^ KO pigs possess CD3^+^CD25^+^ positive cells as well as CD3^+^FOXP3^+^, and CD25^+^FOXP3^+^ DN cells in the thymus, or CD20^+^CD79a^+^ cells in the spleen, indicating that they might have γδ T-cells, or functional B-cells. A previous study showed that FOXP3 positive cells in mice were CD25-positive among DN cells [27], indicating that CD25 expression positively correlated with Foxp3. In this study, however, CD25-positive cells in pigs are partially co-localized with FOXP3-positive staining (Supplementary Figure 3.3). Further, the findings from our present study showed that *mIL2RG*^+/Δ69-368^ KO pigs possess CD3 positive cells as well as CD44^−^CD25^+^ cells (DN3 stage), but lacked CD44^−^CD25^−^ cells (DN4 stage). This is the first critical difference between the pig and mouse immune system.

T-cell development in *mIL2RG* KO pigs successfully progressed to the DN3 stage. This may be associated with different IL2RG/JAK3 signals or the relatively abundant CD3-positive cells. The presence of CD3 on the surface of T-cells depends on the proper rearrangement and functional pairing of T cell receptor (TCR) alpha/beta or gamma/delta chains [27, 28]. The expression of CD4 and CD8 is induced following the pairing of a surrogate alpha with a correctly rearranged beta chain. The appearance of pre-TCR also stops gamma chain rearrangement in T-cells and induces the formation of the alpha chain. Since CD3-positive cells are present in DN T-cells from *mIL2RG* KO pig-derived thymus and spleen, they are likely gamma/delta T cells. Thus there are clear phenotypic differences between *mIL2RG*^+/Δ69-368^ KO pigs and Il2rg mutant mice.

It is well-known that *T-bet* and *GATA-3* in mice are key transcription factors involved in the control of Th1 and Th2 cytokine production [29, 30]. In DNA chip analysis, mRNA expression of *T-bet* and *GATA-3* in the spleen or thymus from *mIL2RG*^+/Δ69-368^ KO pigs was decreased compared with that from WT. However, *STAT-1* and/or *STAT-6*, exist upstream of *T-bet* and *GATA-3*, were expressed at higher levels in spleen cells from *mIL2RG*^+/Δ69-368^ KO pigs compared with those from WT or were not altered, respectively. These observations suggest that the pig IL2RG signalling pathway does not mimic the mouse Il2rg signalling pathway. In addition, the levels of Th1 cytokines, such as IFN-γ, in the spleen or thymus from *mIL2RG*^+/Δ69-368^ KO pigs were significantly higher than those in WT. Because the degree of change in expression was profound for most of these genes (≥2-fold), these findings reinforce the notion that the effect of IL2RG deficiency on T, B, and NK cell development is combinatorial, affecting different signalling pathways involved in many aspects of cellular function.

Recently, several groups have reported the development of X-linked SCID-like pigs, which were created by disrupting the *IL2RG* gene, and used to test allogeneic engraftment [18, 19]. However, limited engraftment was obtained following xenotransplantation of human BM cells to porcine^−/Y^ recipients [18]. Very recently, we successfully transplanted human inducible pluripotent stem cells or porcine trophoblast stem cells into biallelic RAG2 SCID pigs, and produced teratoma from allogenic or xenogeneic cells [21]. Thus xenografted cells can differentiate along a multitude of differentiation pathways, representing a major advance over previous large animal SCID models. Previously, we and other group found that *RAG2* KO pigs lack CD3^+^ cells in their spleen and thymus [20, 21]. On the contrary to this, *mIL2RG*^+/Δ69-368^ KO pig-derived thymus and spleen exhibited several different types of CD3^+^CD4^−^CD8^−^ or CD3^+^CD44^+^CD25^+^, CD3^+^CD44^−^CD25^+^, and FOXP3^+^CD3^+^CD25^+^ cells, indicating that the importance of CD3^+^ T cells against xenotransplantation might be underscored. In conclusion, our observations suggested that functional innate immunity in *IL2RG* KO pigs impedes efficient immunological adaptation of human engraftment and that results of the present study will offer further mechanistic insights into the immune dysfunction of *mIL2RG*^+/Δ69-368^ KO pigs.

## MATERIALS AND METHODS

### Ethics statement

Animals used in this study were approved by the Institutional Animal Care and Use Committee of the University of Missouri (IACUC 7868) and Konkuk University (IACUC KU15085). Tissue collection (Lymph node, spleen, and thymus) was conducted at slaughter according to relevant national and international guidelines.

### Gene targeting

For gene targeting, 2-3 million cells were transfected with TALEN constructs with a reporter vector; 2 μg of each construct was used per 1 million cells. Cells were electrophoresed with the constructs at 490 V for 1 ms and in three pulses using a BTX Electro Cell Manipulator (Harvard Apparatus, Holliston, MA). The cells were plated in T75 flasks for 48 h and sorted for GFP positive cells using Beckman Coulter MoFlo XDP. The sorted cells were plated in 96-well plates. After 10 days, half of the cells were used for genotyping. To investigate the presence of indels following the introduction of TALENS, a fragment of genomic DNA flanking the TALEN cutting site was amplified by PCR. Genomic DNA from cultured cells was isolated using cell lysis buffer, and the genomic DNA was used for PCR. PCR conditions were as follows; initial denaturation for 2 min at 95°C followed by 32 cycles of 30 s at 94°C for denaturation, 30 s at 55°C for annealing, and 30 min at 72°C. The predicted size of the PCR product was 417 bp. RT-PCR products to identify the presence of indels were analysed by 1% of agarose gel electrophoreses, extracted by agarose gel, and then sequences directly by using an automated sequencer, ABI 3730XBI (Applied Biosystems, Foster City, CA, USA).

### Somatic cell nuclear transfer

To produce SCNT embryos, sow-derived oocytes were purchased from ART (Madison, WI). The oocytes were shipped overnight in maturation medium (TCM199 with 2.9 mM Hepes, 5 μg/mL insulin, 10 ng/mL EGF, 0.5 μg/mL p-FSH, 0.91 mM pyruvate, 0.5 mM cysteine, 10% porcine follicular fluid, 25 ng/mL gentamicin) and transferred into fresh medium after 24 h. After 40-42 h of maturation, cumulus cells were removed from the oocytes by vortexing in the presence of 0.1% hyaluronidase. During manipulation, oocytes were placed in manipulation medium supplemented with 7.0 μg/mL cytochalasin B. The polar body, along with a portion of the adjacent cytoplasm, presumably containing the metaphase II plate, was removed and a donor cell was placed in the perivitelline space using a thin glass capillary. The reconstructed embryos were then fused in fusion medium (0.3 M mannitol, 0.1 mM CaCl_2_, 0.1 mM MgCl_2_, and 0.5 mM Hepes) by two DC pulses (1-s interval) at 1.2 kV/cm for 30 μs using a BTX Electro Cell Manipulator (Harvard Apparatus). After fusion, embryos were fully activated with 200 μM thimerosal for 10 min in the dark and 8 mM dithiothreitol for 30 min. Embryos were then incubated in porcine zygote media 3 (PZM3) with 0.5 μM scriptaid, a histone deacetylase inhibitor, for 14-16 h. The following day, the SCNT embryos were transferred into surrogates. To transfer blastocysts, the embryos were washed and cultured for a further 5 days in PZM3 in the presence of 10 ng/mL CSF2. SCNT embryos were surgically transferred into the ampullary-isthmic junction of a surrogate.

### Identification of off-target sequences

To identify putative off-target sequences for the TALENs used in this study, bioinformatics tools were used to identify sequences similar to each TALEN binding site from the most recent pig genome assembly (Sscrofa10.2). PCR primers were designed to flank the most likely off-target sites based on the number of nucleotide differences. These regions were amplified in the founder animals and tested for off-target events using the Surveyor nuclease assay (Supplementary Figure 2). After PCR amplification, 300-500 ng of PCR products (10-15μL) was transferred to a fresh tube, denatured, and re-annealed according to the following thermocycler program: 95°C for 2 min, 95-85°C at −2°C per second, 85-25°C at −0.1°C per second, 4°C indefinitely). One-microliter of Surveyor nuclease and 1 μL of Surveyor enhancer were added and samples were incubated at 42°C for 30 min. Next, the reaction mixtures were immediately placed on ice and 6X Surveyor nuclease stop buffer and 6X dye were added. The samples were electrophoresed on a 2.0% agarose gel.

### Real-time quantitative RT-PCR analysis

The total RNA obtained from each tissue was reverse-transcribed using the QuantiTect Reverse Transcription Kit (Qiagen, USA). To assess levels of gene expression, quantitative real-time reverse transcriptase polymerase chain reaction (RT-qPCR) was conducted using an ABI ViiA 7 system (Applied Biosystems, CA, USA) and SYBR Green as the double-stranded DNA-specific fluorescent dye (Bio-Rad, CA, USA) (Supplementary Table 2, 3, and 5 for RT-qPCR primer sets). GAPDH was used as an internal control to normalise the RT-qPCR efficiency and to quantify gene expression. After normalisation, we compared the relative expression of each mRNA in IL2RG KO pig with that in WT pigs. We performed RT-qPCR on each sample independently and in triplicate.

### Western blot analysis

Tissues from WT and IL2RG KO pigs were lysed in lysis buffer. Equal amounts of protein were resolved by 10% SDS gel electrophoresis, and proteins were electrophoretically transferred to a nitrocellulose membrane. Immunoreactivity was detected through sequential incubation with horseradish peroxidase (HRP)-conjugated secondary antibodies and enhanced chemiluminescence reagents. The primary antibodies used are detailed in Supplementary Table 4.

### Immunohistochemistry

Tissues were fixed in neutral buffer containing 10% formalin and slides were generated for immunohistochemical analysis. Endogenous peroxidase activity was blocked using 3% hydrogen peroxidase following which the samples were pre-treated with Borg Decloaker, and blocked in background Sniper solution. After washing, samples were incubated with primary antibodies specific for either B, T, or NK cells (Supplementary Table 4). After incubation, samples were washed and incubated further with HRP-conjugated secondary antibodies. The samples were also stained with hematoxylin to provide background staining.

### RNA amplification, labelling, and hybridisation to Agilent microarrays

RNA was extracted from the spleen and thymus of *mIL2RG*^+/Δ69-368^ KO and WT pigs using RNeasy Mini Kits (Qiagen, Valencia, CA, USA) according to the manufacturer protocol. We used the Porcine (V2) Gene Expression Microarray, 4×44K (G2519F). Labelling was carried out using an RNA Fluorescent Linear Amplification Kit (Agilent Technologies, Palo Alto, CA, USA; http://www.agilent.com). The sample and control RNAs were labelled with Cy-3 and Cy-5, respectively. Fragmentation was carried out by incubation at 60°C for 30 min in fragmentation buffer (Agilent Technologies), and the process was stopped by the addition of an equal volume of hybridisation buffer (Agilent Technologies). The fragmented target was applied to an Oligo Microarray for pig (Agilent Technologies). Hybridisation was carried out at 60°C for 17 h in a hybridisation oven (Robbins Scientific, Sunnyvale, CA, USA). The hybridised array was scanned with an Agilent microarray scanner. The TIFF image generated was loaded into Feature Extraction Software (Agilent Technologies) for feature data extraction, and data analyses were performed with GeneSpring 10.0. Microarray data have been deposited in NCBI's Gene Expression Omnibus and are accessible through GEO Series accession number GSE77581.

### Pathway studio analysis

To identify molecular pathways, we arranged the data using Pathway Studio 9.0 software (Ariadne Genomics; MD, USA). This program integrates relevant information between the imported genes, consequently permitting the identification of biological pathways, gene regulation networks, and protein interaction maps.

### Gene ontology analysis

Gene ontology (GO) analysis of the significant probe list was performed in PANTHER (http://www.pantherdb.org/), using text files containing the Gene ID list and accession numbers of the Illumina probe ID. All data analysis and visualisation of differentially expressed genes were conducted using R 2.4.1 (www.r-project.org). In addition, DAVID Functional Annotation Bioinformatics Microarray Analysis tools (http://david.abcc.ncifcrf.gov/) were used to study the biological function of the regulated genes [23].

### Kyoto encyclopedia of genes and genomes pathway analysis

Kyoto encyclopedia of genes and genomes (KEGG) is a collection of online databases dealing with genomes, enzymatic pathways, and biological chemicals. The PATHWAY database records networks of molecular interactions in cells, which include organism-specific network maps (http://www.genome.jp/kegg/).

### Statistical analysis

All experimental data was presented as means ± standard deviation (SD). Each experiment was performed at least three times. For statistical analysis, one-way analysis of variance (ANOVA) was performed to determine whether there were differences among the groups, and Fisher's post-test was performed to determine significance between pairs of groups. In all experiments, *P < 0.05, and **P < 0.01 were considered significant.

## SUPPLEMENTARY MATERIALS FIGURES AND TABLES




