# Supplementary material for: Partial loss of interleukin 2 receptor gamma function in pigs provides mechanistic insights for the study of human immunodeficiency syndrome
Source: Oncotarget. 2016 Jul 24;7(32):50914–26. doi: 10.18632/oncotarget.10812 (PMC5239447; doi:10.18632/oncotarget.10812)
Supplement: Supplementary file 1 [file oncotarget-07-50914-s001.pdf]

# Partial loss of interleukin 2 receptor gamma function in pigs provides mechanistic insights for the study of human immunodeficiency syndrome

**Supplementary Data**

**Supplementary Table S1: Production of IL2RG pig**

| No of Experiments | Numbers of embryos transferred | No of IL2RG Piglets |
|---|---|---|
| **#1** | 216 | 1 |
| **#2** | 48 Blastocysts | 4 |
| **#3** | 187 | 2 |
| **#4** | 179 | 4 |

**Supplementary Table S2: Primer set lists for IL2RG KO pig analysis**

| Gene | | Primer sequences |
|---|---|---|
| **IL2RG** | Forward | CTGGACTATTAGAAGGATGTGGGC |
| | Reverse | ATATAGTGGGAAGCCTGGGATGGCT |

*PCR product size: 225bp and 192bp

**Supplementary Table S3: Primer set lists for off-targeting analysis of IL2RG KO pig**

| Gene | | Primer sequences |
|---|---|---|
| **LRRIQ1** | Forward | CGTTTGTTAAAACTGCAGCATA |
| | Reverse | TTTTGCTTCCCTTTCCTTCC |
| **BNC2** | Forward | AGCCAGAGGAAGGGGTTTTA |
| | Reverse | GTTAACCAGCTCAGGCAAC |
| **SLC17A5** | Forward | GTCTGGTTGCAGCTCAAGGT |
| | Reverse | GCCACTGTGGACTCTAGAGGAT |
| **ZNF334** | Forward | ATTCACACAGGGGAGAAACG |
| | Reverse | GTGGAAATTTTTCCCCCATT |
| **TTN** | Forward | CTTTGGACCTGCCCACTTT |
| | Reverse | GGATGTGTGATCGGTTCCAT |
| **PGRMC2** | Forward | TGAGGGAGAGAGGAGACCTG |
| | Reverse | CTAGGGGAAGGAAAGGGATG |
| **AVPR2** | Forward | GGCGTACATGCCTACCATCT |
| | Reverse | CTGTCCACGGTCTTTGTGG |
| **CCDC18** | Forward | TTCTCCCAACCCCATTTACA |
| | Reverse | CCTGAGTTGAACCAGCACCT |
| **ZSWIM2** | Forward | AAAAAGTTCTTCCTGTTTTGACAGA |
| | Reverse | TGGTTATTCCACCAATGCAA |

**Supplementary Table S4: Antibody list for immunohistochemistry**

| Primary antibodies | Host | Dilution | Source |
|---|---|---|---|
| **IL-2Rα** | Mouse | 1/100 | Santa Cruz Biotechnology, |
| **IL2Rγ** | Rabbit | 1/200 | Bioss |
| **CD3** | Mouse (LN10) | 1/50 | Leica Biosystems |
| **CD4** | Rabbit | 1/100 | LSbio |
| **CD8** | Goat | 1/100 | LSbio |
| **CD20** | Mouse(MJ1) | Predilution | Leica Biosystems |
| **CD21** | Mouse(2G9) | Predilution | Leica Biosystems |
| **CD79a** | Mouse(11E3) | Predilution | Leica Biosystems |
| **CD56** | Mouse(1B6) | 1/100 | Novocastra |
| **CD25** | Mouse(4C9) | predilution | Leica Biosystems |
| **CD1d** | Mouse | 1/100 | Santa Cruz Biotechnology, |
| **CD44** | Mouse | 1/100 | LSbio |
| **CD335** | Rabbit | 1/100 | Bioss |
| **FOXP3** | Rabbit | 1/500 | Abcam |

**Supplementary Table S5: Primer list for Real-Time RT-PCR**

For Table S5, please see the attached Excel file

**Supplementary Table S6: KEGG in thymus**

| KEGG | Genes |
|---|---|
| Cytokine-cytokine receptor interaction | CCL2, IL6ST, IL18, CXCL2, CXCL9, CCL8, IL15, CCL5, CCL4, CXCL12, IL10, CXCL10, IL12RB1, IL10RA, IFNG, CXCR6, TPO, IL2RG, FAS, EGF, IL13RA1, IFNGR2, IL1A, GHR, IL4, EGFR, MET, LIFR, CCL19, CD40, KDR, CCR9, ACVR2A, TNFSF10, CCR6, TNFSF13B, VEGFA, BMP7, BMPR1A |
| Jak-STAT signaling pathway | IL4, IL6ST, LIFR, BCL2L1, IL15, STAT1, IL10, IL12RB1, CCND3, IL10RA, IFNG, TPO, IL2RG, IL13RA1, IFNGR2, GHR |
| Leukocyte transendothelial migration | F11R, CLDN7, OCLN, CLDN4, MMP9, MYLPF, CLDN10, CXCL12, CDH5, VCL, MYL9, VCAM1, MAPK12, MAPK13, MLLT4 |
| Complement and coagulation cascades | PLAT, A2M, C3, CFB, SERPING1, C1S, C1QC, C1QA, VWF, C1QB, THBD, SERPINE1, CFH, C2 |
| Hematopoietic cell lineage | IL4, CD9, CD36, CD3G, CD3D, CD8A, CD3E, CD2, TPO, ANPEP, CD4, ITGB3, IL1A |
| Systemic lupus erythematosus | C1QA, C1QB, CD80, FCGR2B, C3, IFNG, H2AFZ, C1S, CD40, C2, C1QC, IL10 |
| T cell receptor signaling pathway | IL4, CD3G, MAPK12, CD8A, CD3D, CD3E, MAPK13, LCK, IFNG, CD247, CD4, IL10 |

| | |
|---|---|
| TGF-beta signaling pathway | ACVR2A, ID1, SMAD7, SMAD6, IFNG, ID4, DCN, BMP7, THBS1, BMP5, BMPR1A |
| Hypertrophic cardiomyopathy (HCM) | SLC8A1, ACTC1, CACNA2D1, TNNC1, CACNB1, IGF1, SGCD, ITGB3, PRKAA2, TPM1 |
| Aldosterone-regulated sodium reabsorption | ATP1B1, NR3C2, HSD11B2, IGF1, IGF2, SFN, IRS1, SLC9A3R2 |
| Intestinal immune network for IgA production | CCR9, IL4, TNFSF13B, CD80, IL15, CD40, CXCL12, IL10 |
| Primary immunodeficiency | CD8A, CD3D, CD3E, LCK, IL2RG, CD4, CD40 |
| Allograft rejection | IL4, PRF1, CD80, IFNG, FAS, CD40, IL10 |
| Glycosphingolipid biosynthesis | B3GALNT1, HEXB, FUT1, FUT2 |

**Supplementary Table S7: Cellular component in thymus**

For Table S7, please see the attached Excel file

**Supplementary Table S8: Molecular function in thymus**

| Molecular function | Genes |
|---|---|
| Identical protein binding | CLDN7, CLDN4, CD8A, TNNC1, IL6ST, HEXB, SNCA, E2F8, TPD52, HOOK1, APP, APOA1, PLOD1, FAP, FAS, GHR, EGFR, MLXIPL, C1QB, VEGFA, COL1A2, NPPC, COL1A1, CAV2, CAV1, ENPP1, CD247, CLDN10, BCL2L1, ITGB3, CD74, SLC11A1, IGF1R, CD2, CD4, THBS1, CEBPA, S100A16, SMAD6, EPHX2, MUL1, CIDEA, CENPF, BIRC5, ADIPOQ, VWF, FAAH, HPGD, BMPR1A |
| Protein kinase activity | FGFR2, CAV2, CCL2, NEK2, PKMYT1, AURKA, AURKB, PRKG1, CCL4, IGF1R, SBK1, TEK, MASTL, PRKAA2, SYK, SRPK3, EGFR, CDK1, BCR, MET, PDK4, AXL, NPR1, PBK, IRS1, WEE1, KDR, EPHA4, ACVR2A, CAMK4, MAPK12, CCND3, MAPK13, PLK1, NTRK2, LCK, GRK5, CIT, MELK, CDC42BPB, BMPR1A |
| Protein homodimerization activity | CAV2, CD8A, ENPP1, TNNC1, IL6ST, HEXB, CD247, E2F8, TPD52, SLC11A1, PLOD1, FAP, CD2, CD4, GHR, CEBPA, S100A16, SMAD6, EPHX2, MLXIPL, CIDEA, CENPF, BIRC5, ADIPOQ, VWF, C1QB, FAAH, VEGFA, NPPC, HPGD, BMPR1A |

| | |
|---|---|
| Cytokine activity | IL4, CCL2, IL18, CXCL2, CXCL9, CCL8, CCL19, IL15, CCL5, ADIPOQ, CXCL12, CCL4, IL10, CXCL10, TNFSF10, TNFSF13B, VEGFA, IFNG, BMP7, IL1A, BMP5, CMTM5 |
| Cytokine binding | A2M, IL6ST, LIFR, CD74, CCR9, CCR6, IL12RB1, CD36, DARC, IL10RA, CXCR6, IL2RG, IL13RA1, THBS1, IFNGR2, GHR, CCRL1 |
| Polysaccharide binding | FGFR2, SELP, CCL2, ENPP1, TLR2, PGLYRP1, CCL8, FGF10, POSTN, DCN, COL5A3, TINAGL1, APP, VEGFA, ADAMTS1, THBS1, BMP7 |
| Glycosaminoglycan binding | FGFR2, SELP, CCL2, TLR2, PGLYRP1, CCL8, FGF10, POSTN, DCN, COL5A3, APP, VEGFA, ADAMTS1, THBS1, BMP7 |
| Extracellular matrix structural constituent | AMBN, LAMA4, COL27A1, LUM, COL1A2, COL15A1, MGP, CD4, COL1A1, TINAGL1, COL5A3, LAMB1 |
| Chemokine activity | CCL2, CXCL2, CXCL9, CCL8, CCL19, CCL5, CCL4, CXCL12, CXCL10 |
| Insulin-like growth factor binding | IGF1R, WISP3, IGFBP7, KAZALD1, IGF2, IGFBP3, IGFBP5 |

**Supplementary Table S9: Biological function in thymus**

| Biological function | Genes |
|---|---|
| Immune response | CD8A, IL18, SNCA, PPARG, TLR2, PGLYRP1, TLR3, TLR4, IL15, CXCL12, C1QC, IL10, CXCL10, CFP, IFNG, CFH, LTF, MS4A2, FAS, IL1A, CCRL1, CRISP3, SIT1, SERPING1, PDCD1LG2, CCR9, C1QA, C1QB, CCR6, TNFSF13B, VEGFA, SEMA4D, GBP1, CCL2, ENPP1, C3, CYSLTR2, CXCL2, CLU, CXCL9, CCL8, C1S, CCL5, CCL4, CD74, IGF1R, SLC11A1, IL2RG, CD4, C2, THBS1, IL4, OLR1, CFB, SMAD6, MYLPF, CCL19, SAMHD1, TINAGL1, CD180, TNFSF10, FCGR2B, CD274, DMBT1, BMPR1A |
| Inflammatory response | A2M, ADORA3, CCL2, S100A8, AIF1, C3, CLU, CXCL2, S100A9, TLR2, CXCL9, TLR3, CCL8, TLR4, IL15, C1S, CCL5, C1QC, CCL4, IL10, CXCL10, CFP, SLC11A1, CFH, ITIH4, MS4A2, C2, THBS1, IL1A, F11R, SELP, OLR1, CFB, EPHX2, CCL19, IGF2, SERPING1, CD40, CDO1, CD180, CD163, C1QA, C1QB, DARC |
| Response to hormone stimulus | ME1, CAV2, RBP4, HMGB2, A2M, CAV1, CCL2, ENPP1, IGFBP7, PTGS1, PPARG, AURKA, BCL2L1, GNG12, CCL5, AQP1, IL10, IGF1R, FAS, THBS1, AGRP, GHR, ACTA1, GATM, CRYAB, MGP, IGF2, NPY1R, CDO1, STAT1, ADIPOQ, IRS1, ABCB4, RERG, C1QB, RETN, CDKN1A, BTG2, FABP3, FABP4, HSD11B2, SORT1, COL1A1, BMP7 |
| Positive regulation of immune system process | EDN3, BLM, IL6ST, C3, IL18, CLU, CD247, TLR2, KLRK1, TLR3, TLR4, IL15, C1S, C1QC, CXCL12, CD74, VCAM1, CFP, SLC11A1, IFNG, CD2, CFH, MS4A2, CD4, IL2RG, C2, IL13RA1, THBS1, LAG3, SYK, IL4, CD3E, CFB, SERPING1, CD40, PDCD1LG2, C1QA, C1QB, CDKN1A, TNFSF13B, CD80, VEGFA, LCK |

| | |
|---|---|
| Regulation of cell activation | BLM, IL6ST, IL18, SNCA, KLRK1, TLR4, IL15, CD74, IL10, VCAM1, IFNG, CD2, MS4A2, CD4, IL2RG, FAS, THBS1, IL13RA1, LAG3, SYK, IL4, SELP, SIT1, CD3E, CD40, PDCD1LG2, CDKN1A, TNFSF13B, CD80, CD274, LCK |
| Blood circulation | CAV2, EDN3, CAV1, TNNC1, MYL1, PPARG, PTGS1, CXCL12, TPM1, CXCL10, EDNRA, EDNRB, CALCB, GUCY1A3, ACTC1, OLR1, ACTA2, EPHX2, NPR1, SERPING1, NPY1R, STAT1, ADIPOQ, NPY, PLN, VEGFA, COL1A2, NPPC, ACE2, HSD11B2, GUCY1B3 |
| Regulation of leukocyte activation | BLM, IL6ST, IL18, SNCA, KLRK1, IL15, CD74, IL10, VCAM1, IFNG, CD2, MS4A2, CD4, IL2RG, FAS, THBS1, IL13RA1, LAG3, SYK, IL4, SIT1, CD3E, CD40, PDCD1LG2, CDKN1A, TNFSF13B, CD80, CD274, LCK |
| Positive regulation of leukocyte activation | IL4, BLM, IL6ST, CD3E, IL18, KLRK1, IL15, CD40, PDCD1LG2, CD74, VCAM1, CDKN1A, TNFSF13B, CD80, LCK, IFNG, CD2, MS4A2, CD4, IL2RG, THBS1, IL13RA1, SYK |
| Positive regulation of phosphorylation | EGFR, IL4, CAV1, IL6ST, CD3E, IGF1, IGF2, TLR4, ADIPOQ, EDNRA, EDNRB, ACVR2A, CD80, CCND3, IFNG, CD4, BMP7, THBS1, EGF, SYK, BMPR1A, GHR |

**Supplementary Table S10: KEGG in spleen**

| KEGG | Genes |
|---|---|
| Cytokine-cytokine receptor interaction | TNF, IL8, CXCL9, CD70, IL15, CXCL11, CCL4, CXCL10, TNFSF10, TNFSF13B, CCR5, IL10RB, SR-PSOX, IFNG, IL1B, EPOR, IL12B |
| Chemokine signaling pathway | CCL2, CCR5, IL8, SR-PSOX, CCL21, CXCL9, STAT1, CXCL11, LOC780409, CCL4, CXCL10 |
| Chemokine signaling pathway | CCL2, CCR5, IL8, SR-PSOX, CCL21, CXCL9, STAT1, CXCL11, LOC780409, CCL4, CXCL10 |
| NOD-like receptor signaling pathway | NOD2, CCL2, TNF, NOD1, IL8, IL1B, CASP1 |
| Systemic lupus erythematosus | TNF, C3, FCGR1A, IFNG, LOC396781, C1S, C2 |
| Allograft rejection | TNF, SLA-3, IFNG, LOC396781, IL12B, SLA-5 |
| Type I diabetes mellitus | TNF, SLA-3, IFNG, IL1B, IL12B, SLA-5 |
| Allograft rejection | TNF, SLA-3, IFNG, LOC396781, IL12B, SLA-5 |
| Jak-STAT signaling pathway | IL10RB, IFNG, EPOR, IL15, IL12B, STAT1 |
| Graft-versus-host disease | TNF, SLA-3, IFNG, IL1B, SLA-5 |
| Viral myocarditis | ITGAL, SLA-3, LOC396781, SLA-5 |
| Complement and coagulation cascades | C3, BDKRB1, C1S, C2 |
| Autoimmune thyroid disease | SLA-3, LOC396781, SLA-5 |

**Supplementary Table S11: Cellular component in spleen**

| Cellular component | Genes |
| --- | --- |
| extracellular region | CCL2, TNF, C3, CXCL9, CD70, LOC733603, IL15, CXCL11, CCL4, CXCL10, TIMP1, NPG4, CCL21, IFNG, IL1B, TGFA, C2, LOC396596, IL8, IL27, PMAP-23, CD163, VWF, AMBN, ADM, NPY, SR-PSOX, SLPI, EGFL8, IL12B, IGFBP3, LOC780409 |
| extracellular region part | CCL2, TNF, IL8, C3, IL27, LOC733603, CD70, IL15, CXCL11, CCL4, TIMP1, VWF, AMBN, NPY, CCL21, SR-PSOX, IFNG, TGFA, IL1B, IL12B, LOC780409 |
| intrinsic to membrane | ITGAL, TLR10, TNF, TBXAS1, SLC15A2, SLC7A9, BDKRB1, CD70, GP91-PHOX, AQP1, SLA-5, CD163, SLA-3, CCR5, TFRC, SR-PSOX, TAP1, CD274, TGFA, EPOR, TREM1 |
| extracellular space | CCL2, TNF, IL8, C3, IL27, LOC733603, CD70, IL15, CXCL11, CCL4, NPY, CCL21, SR-PSOX, IFNG, IL1B, TGFA, IL12B, LOC780409 |
| plasma membrane | ITGAL, TNF, SLA-3, TFRC, CCR5, IRG6, TGFA, BDKRB1, SLA-5, CD163 |
| membrane-bounded vesicle | VWF, TFRC, ESD, CTSB |
| cytoplasmic membrane-bounded vesicle | VWF, TFRC, ESD, CTSB |
| vesicle | VWF, TFRC, ESD, CTSB |
| cytoplasmic vesicle | VWF, TFRC, ESD, CTSB |

**Supplementary Table S12: Molecular function in spleen**

| Molecular function | Genes |
|---|---|
| cytokine activity | CCL2, TNF, IL8, IL27, CXCL9, CD70, IL15, CXCL11, CCL4, CXCL10, CCL21, SR-PSOX, IFNG, IL1B, IL12B, LOC780409 |
| cation binding | ADSSL1, TBXAS1, CYP3A39, C1S, STAT1, GP91-PHOX, CAPN3, SOD2, DDX58, IRG6, EGFL8, KLF1, GBA, ALOX12 |
| ion binding | ADSSL1, TBXAS1, CYP3A39, C1S, STAT1, GP91-PHOX, CAPN3, SOD2, DDX58, IRG6, EGFL8, KLF1, GBA, ALOX12 |
| metal ion binding | DDX58, ADSSL1, TBXAS1, CYP3A39, IRG6, EGFL8, C1S, GP91-PHOX, STAT1, CAPN3, KLF1, ALOX12, SOD2 |
| nucleotide binding | DDX58, IFIH1, ADSSL1, CKM, TAP1, RBM38, OAS1, OAS2, GP91-PHOX, MX1 |
| purine nucleotide binding | DDX58, IFIH1, ADSSL1, CKM, TAP1, OAS1, OAS2, GP91-PHOX, MX1 |
| chemokine activity | CCL2, IL8, CCL21, CXCL9, CXCL11, LOC780409, CCL4, CXCL10 |
| chemokine receptor binding | CCL2, IL8, CCL21, CXCL9, CXCL11, LOC780409, CCL4, CXCL10 |
| transition metal ion binding | DDX58, TBXAS1, CYP3A39, IRG6, GP91-PHOX, KLF1, ALOX12, SOD2 |
| ribonucleotide binding | DDX58, IFIH1, ADSSL1, CKM, TAP1, OAS1, OAS2, MX1 |

**Supplementary Table S13: Biological function in spleen**

| Biological function | Genes |
|---|---|
| immune response | CCL2, TNF, C3, CXCL9, CD70, OAS1, IL15, C1S, OAS2, CXCL11, SLA-5, CCL4, CXCL10, SLA-3, CCL21, IRG6, IFNG, IL1B, C2, TLR10, IL8, IL27, DDX58, TNFSF10, TNFSF13B, LOC780409 |
| defense response | TLR10, CCL2, IL8, C3, IL27, LYZ, PMAP-23, LOC733603, C1S, CCL4, CD163, DDX58, NPG4, IRG6, IL1B, C2 |
| response to wounding | VWF, CCL2, IL8, C3, IL27, IL1B, LOC733603, C1S, C2, CCL4, CD163 |
| inflammatory response | CCL2, IL8, C3, IL27, IL1B, LOC733603, C1S, C2, CCL4, CD163 |
| proteolysis | C3, IL1B, LOC733603, C1S, C2, CD163 |
| proteolysis | C3, IL1B, LOC733603, C1S, C2, CD163 |
| oxidation reduction | CCL2, NPY, IL8, SR-PSOX, CCL4 |
| acute inflammatory response | NOD2, IFIH1, NOD1, CASP1, IGFBP3 |
| behavior | NOD2, IFIH1, NOD1, CASP1, IGFBP3 |
| regulation of cell death | CCL2, IL8, SR-PSOX, CCL4 |

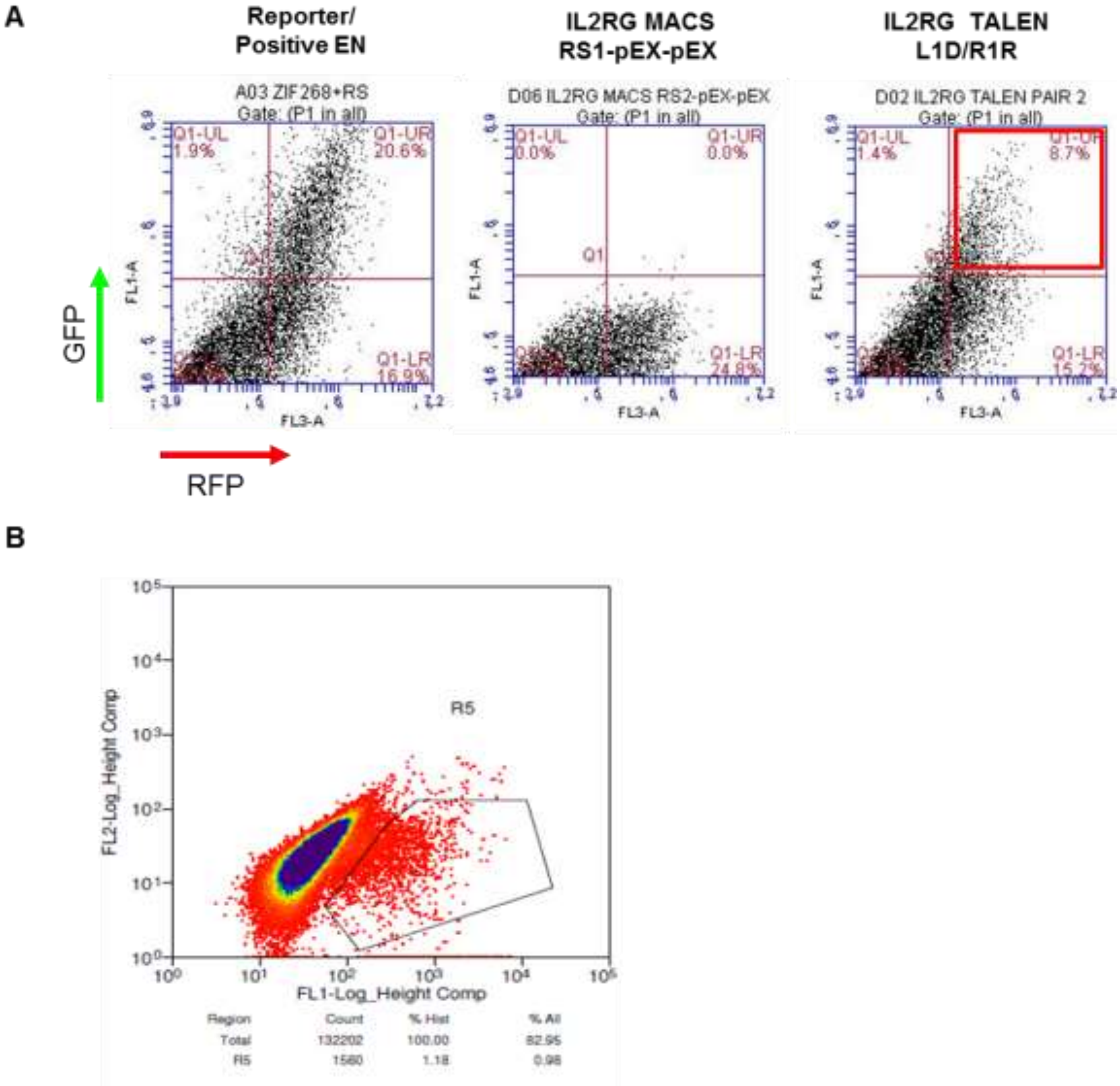


**Supplementary Figure S1. Cell sorting analysis harboring IL2RG mutated gene.** (A) The activity of the designed TALENs was pre-validated by introducing the TALENs with a reporter into HEK 293T cells. Enrichment of cells expressing RFP/GFP, compared to controls of RFP/GFP positive cells, was detected when TALEN sets were introduced with a reporter. After validation, the constructs coding for TALENs and a reporter for IL2RG were introduced into pig fibroblast cells by electroporation (B). After 48h post-transfection, the

Cells were sorted for GFP+ cells by FACS and individually plated into 96-well plates. The frequency of GFP + cells from each transfection ranged from 23.0% to 38.0%. The box blew the arrow indicates the gate used to sort GFP+ cells.

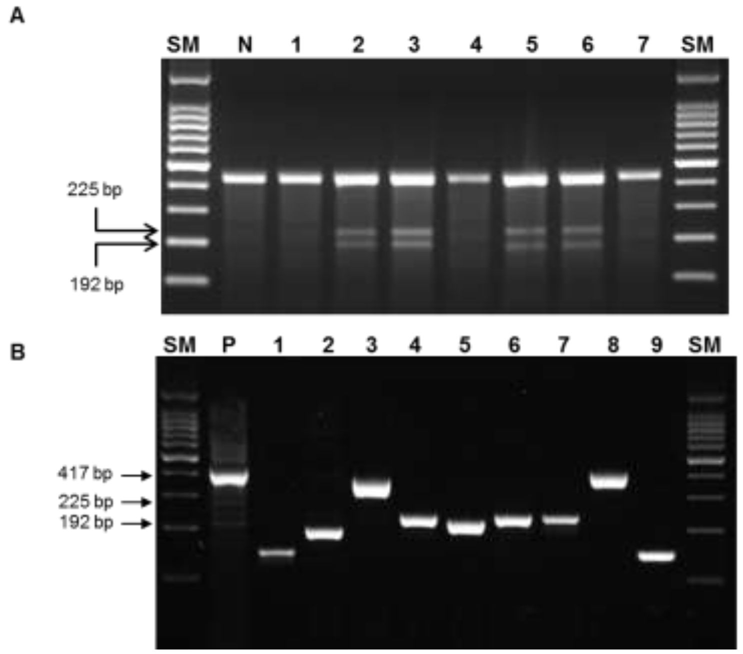


**Supplementary Figure S2. Off-site targeting analysis of IL2RG genes from IL2RG mutated pig.** (A) Genotyping of IL2RG KO piglets (B) Surveyor nuclease digest of heteroduplex DNA revealed no additional off-targetng mutations at the 9 loci with highest homology to pig IL2RG gene; SM: size marker, lane 1:LRRIQ1, 2:BNC2, 3:SLC17A5, 4:ZNF334, 5:TTN, 6:PGRMC2, 7:AVPR2, 8:CCDC18, 9:ZSWIM2

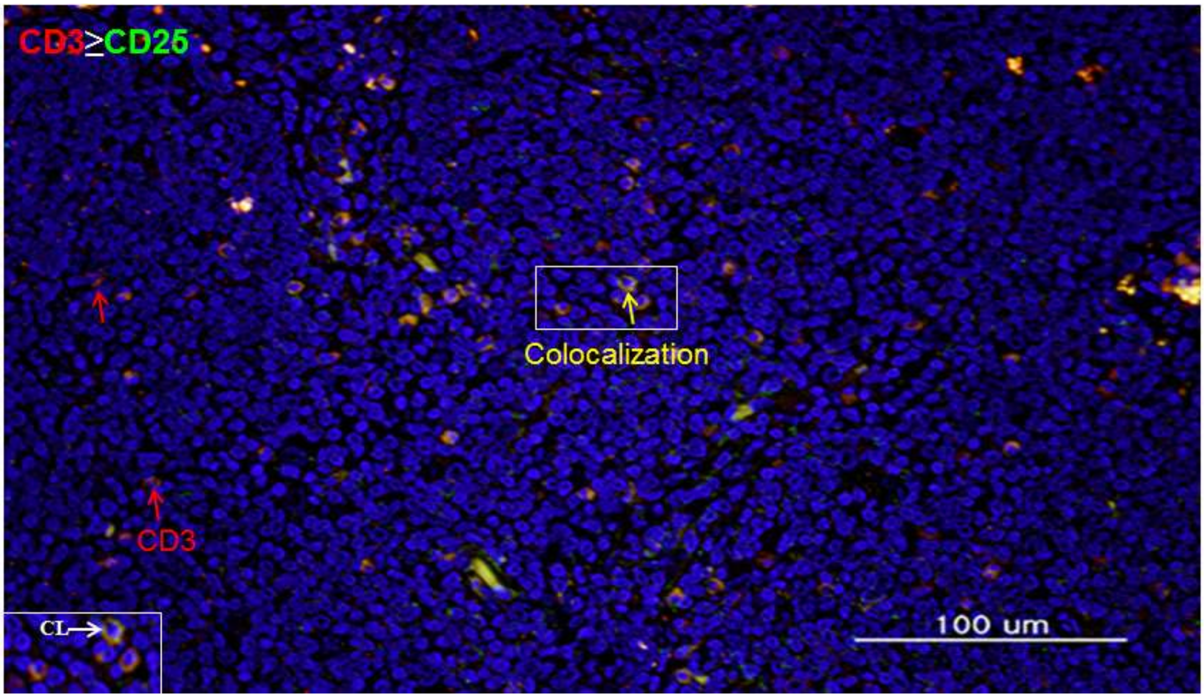


**Supplementary Figure S3.1. Immunohistochemistry analysis of CD3 and CD25 positive cells in thymus.** For co-localization of CD25 and CD3, thymic paraffin sections were stained with CD3 (red) and CD25 (green) antibodies where the DP cells appear yellow in merged images due to the co-expression of CD3 and CD25 proteins. CD3 staining indicates maturing T lymphocytes, whereas CD25 staining shows DN2-3 T cells or Treg cells.

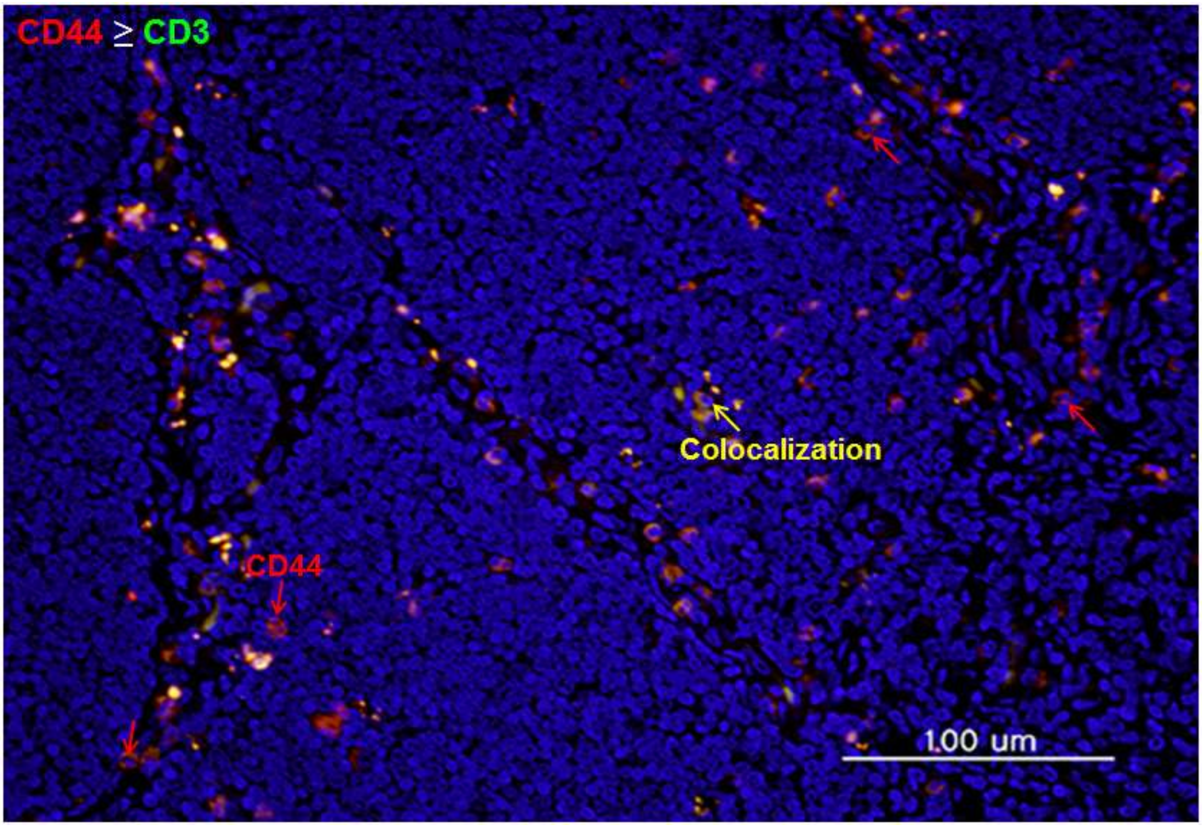


**Supplementary Figure S3.2. Immunohistochemistry analysis of CD3 and CD44 positive cells in thymus.** For co-localization of CD3 and CD44, thymic paraffin sections were stained with CD3 (green) and CD44 (red) antibodies. CD44 staining indicate memory T helper 1 (Th1) cells and DN1-2 stage of T cell development.

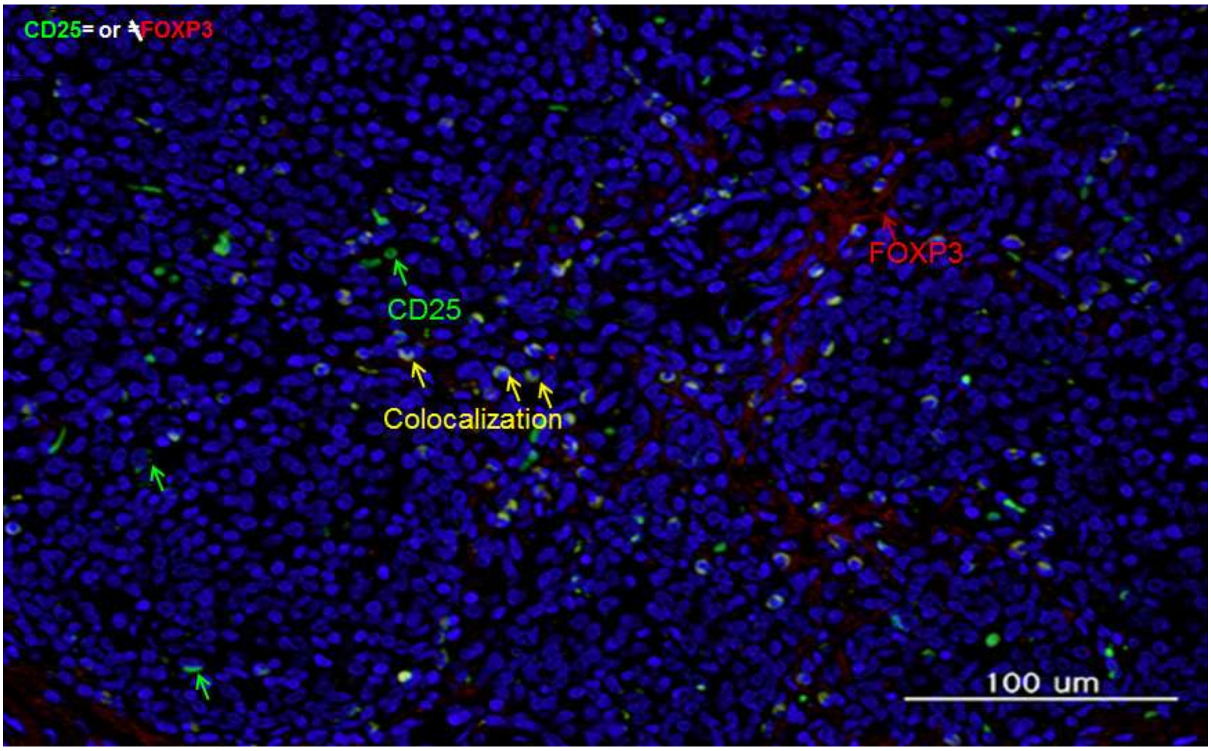


**Supplementary Figure S3.3. Immunohistochemistry analysis of CD25 and FOXP3 positive cells in thymus.** For co-localization of CD25 and FOXP3 expression, thymic paraffin sections were stained with CD25 (green) and FOXP3 (red) antibodies. FOXP3 staining indicates medullary mature Treg.

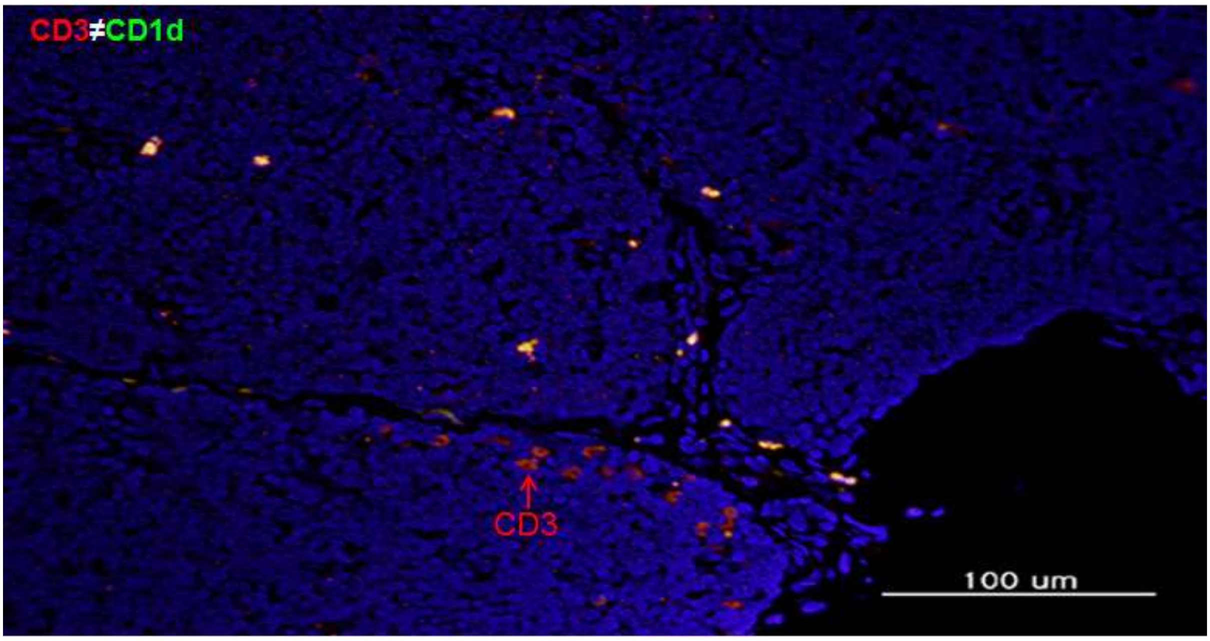


**Supplementary Figure S3.4. Immunohistochemistry analysis of CD3 and CD1d positive cells in thymus**. For co-localization of CD3 and CD1d staining, thymic paraffin sections were stained with CD3 (red) and CD1d (green) antibodies. CD1d-presented lipid antigens indicate natural killer T (NKT) cells.

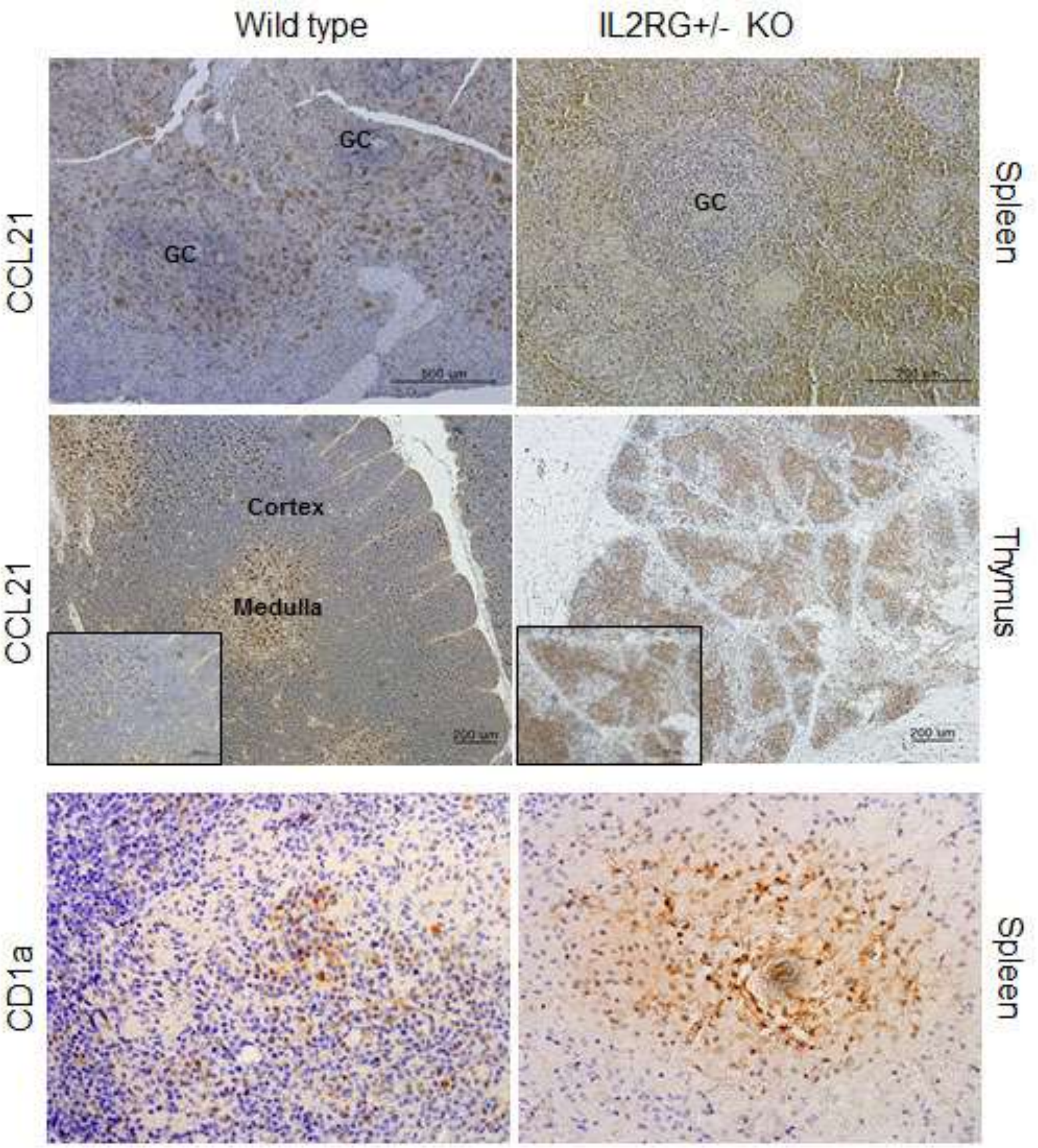


**Supplementary Figure S3.5. Immunohistochemistry analysis of CCL21 and CD1a positive cells in spleen and thymus.** CCL21 and CD1a indicate a maker of immature DC cells.

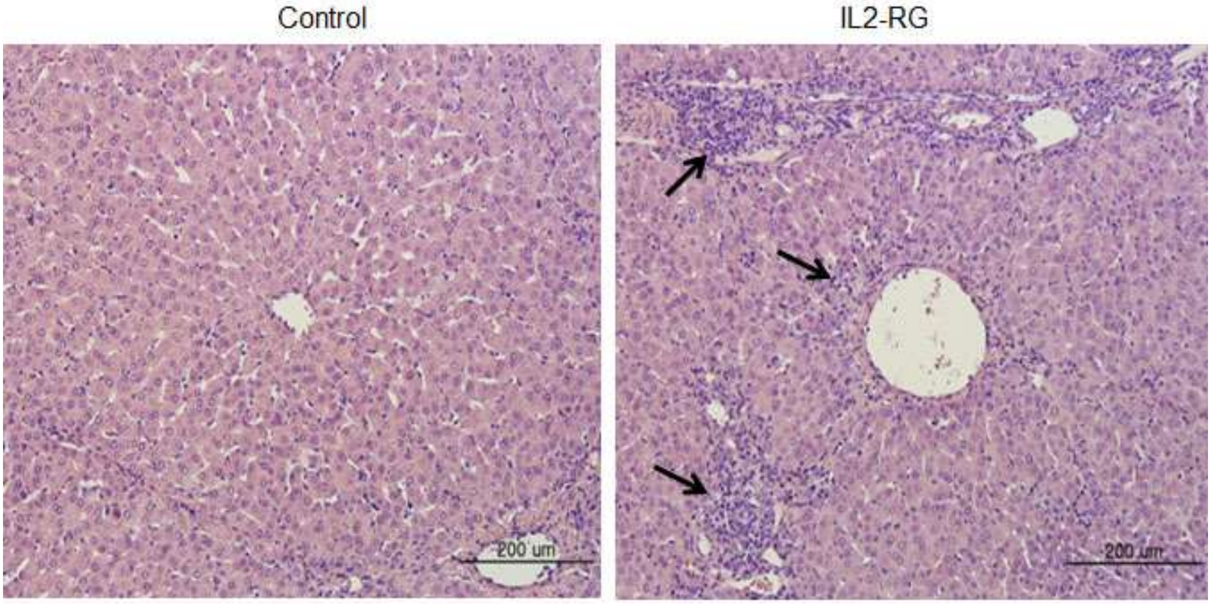


**Supplementary Figure S4. Macrophages infiltration in liver of *mIL2RG*$^{+/\Delta 69\text{-}368}$ KO pig.**

Arrow indicate abnormal lymphocyte infiltration into liver of *mIL2RG*$^{+/\Delta 69\text{-}368}$ KO pig.

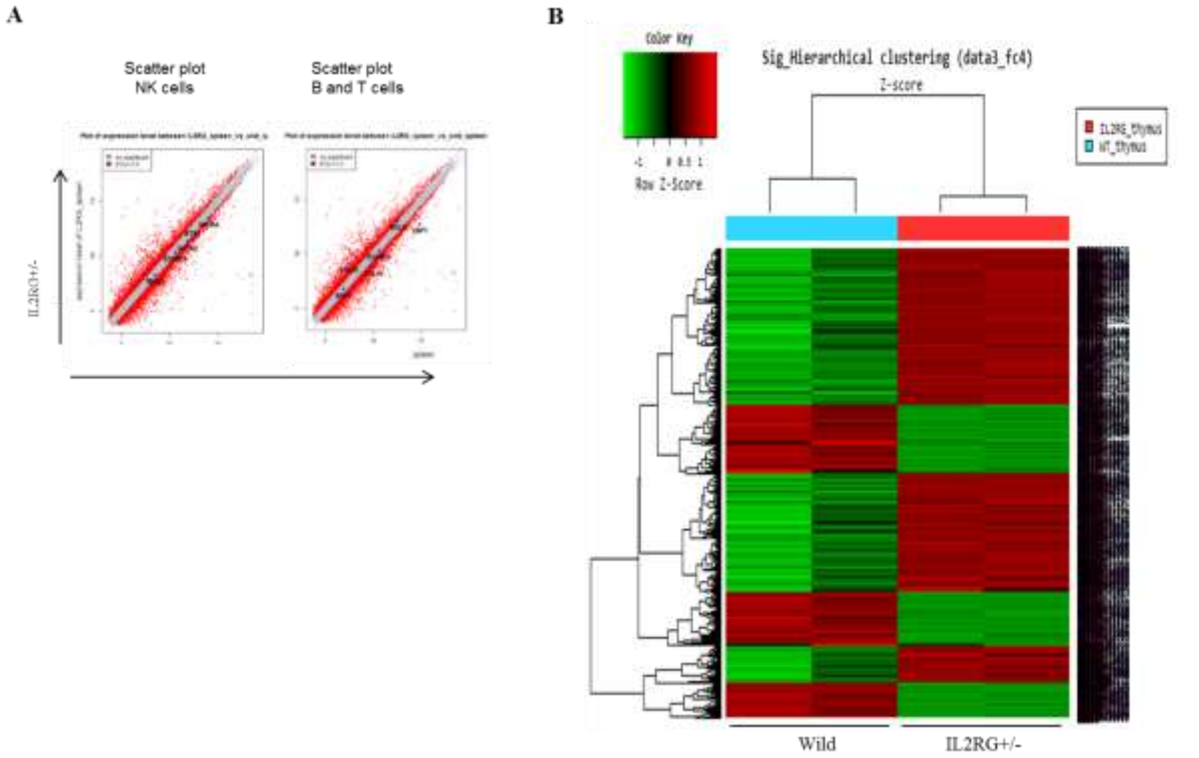


**Supplementary Figure S5. Hierarchical clustering in WT and *mIL2RG*$^{+/\Delta 69\text{-}368}$ KO pig.** Pairwise scatter plot analysis of the global gene expression profiles of NK (left) or B/T cell (right) from IL2RG KO pig and WT. Black lines indicate boundaries of 2-fold difference in gene expression levels. The bar to the right indicates the scattering density; the higher the scattering density, the darker the grey color. Gene expression levels are depicted in $\log_2$ scale. The number of differentially expressed genes is indicated under each scatter plot(A). Hierarchical clustering of WT and IL2RG thymus(B). Color represents the log intensities: Red: upregulated genes; green: downregulated genes. The heat map was plotted using “Heatplus” package in R program.

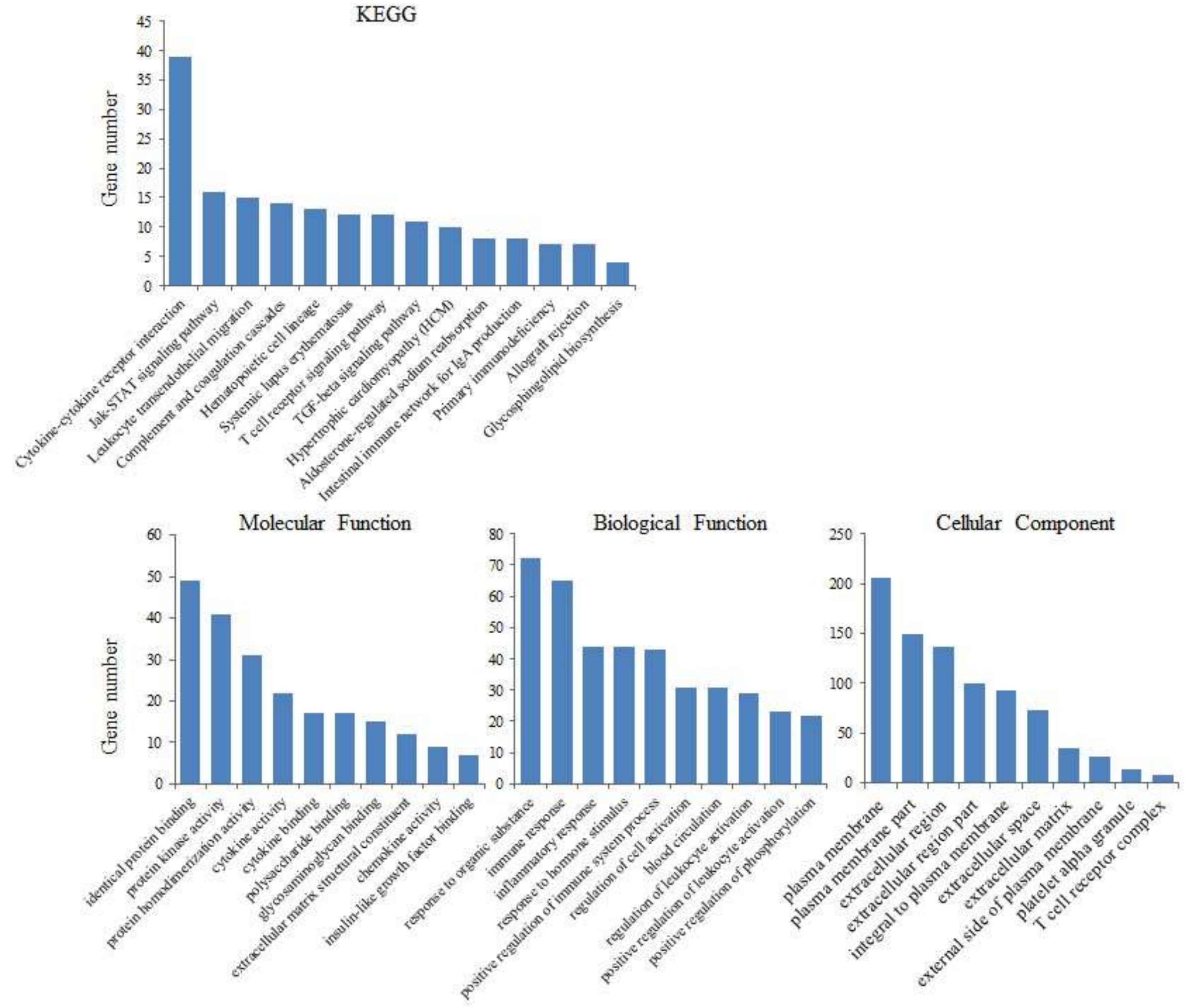


**Supplementary Figure S6**. **KEGG pathway enrichment analysis, GO biology process,** molecular function, and cellular function analysis in WT and mIL2RG$^{+/\Delta 69\text{-}368}$ KO pig thymus. Using DAVID program, up- and down- regulated gene expressions were divided into 3 subgroups such as Biological process, Cellular component, and Molecular function. Each subgroup shows the top 10 of the gene function group.

**A**

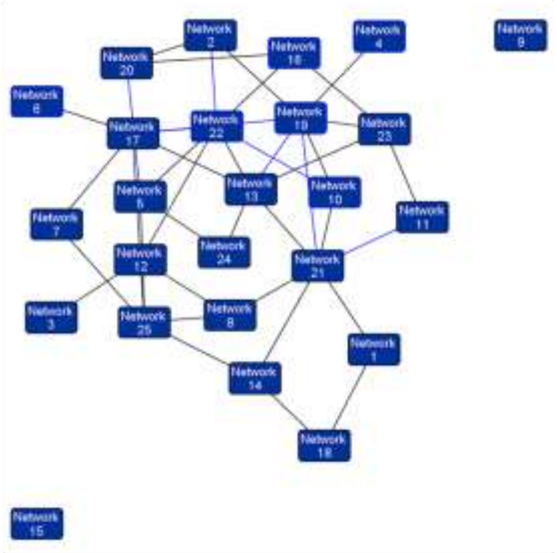


**B**

| ID network | Focus molecule | Score | Top disease and functions |
|---|---|---|---|
| 1 | 29 | 36 | Cardiovascular system development and function, Organismal defvelopment, Organ morphology |
| 2 | 29 | 36 | Cancer, Organismal injury and abnormalities, dermatological disease and conditions |
| 3 | 27 | 32 | Cellular development, Tissue development, Skelectal and muscular system develop ment and function |
| 4 | 26 | 30 | Connective tissue disorders, **immunological disease, inflammaroty disease** |
| 5 | 26 | 30 | Cell-to-cell signaling and interaction, **Hematological system development and fun ction, Immune cell trafficking** |
| 6 | 26 | 30 | Cancer, Organismal injury and abnormalities, Reproductive system disease |
| 7 | 25 | 28 | Cell cycle, DNA replicaion, Recombination and repair, Cell death and survival |
| 8 | 25 | 28 | Cancer, Organismal injury and abnormalities, Reproductive system disease |
| 9 | 23 | 25 | Cell-to-cell signaling and interaction, Cellular function and maintenance, **Hematolo gical system development and function** |
| 10 | 23 | 25 | Gastrointestinal disease, Hepatic system disease, Liver fibrosis |
| 11 | 23 | 25 | Endocrine system Disorders, Gastrointestinal disease, Immunogical disease |
| 12 | 23 | 23 | Carbohydrate metabolism, Lipid metabolism, Small molecular biochemistry |
| 13 | 22 | 21 | Digestive system development and function, Organ morphology, Mervous system de velopment and function |
| 14 | 21 | 21 | Cancer, **Hematological disease, Immunological disease** |
| 15 | 21 | 20 | Connective tissue disorder, **Immunological disease, Inflammatory disease** |
| 16 | 20 | 17 | Cardiac hypertrophy, Cardiovascular disease, Developmental disorder |
| 17 | 18 | 17 | Cellular development, Cancer, hematological disease |
| 18 | 18 | 17 | Cellular assembly and organization, Prgan morphology, Cancer |
| 19 | 18 | 17 | Connective tissue disorders, Dermatological disease and conditions, Developmental disorder |
| 20 | 18 | 17 | Cell death and survival, Cellualr assembly and organization, DNA replication, Reco mbination and repair |
| 21 | 17 | 15 | Cellular movement, Carbohydrate metabolism, Molecualr transport |
| 22 | 17 | 15 | Cardiovascular system development and function, Organ morphology, Behavior |
| 23 | 16 | 14 | Developmental disorder, Hereditary disorder, Ophthalm c disease |
| 24 | 16 | 14 | Cellular compromise, Cellular function and maintenance, cell death and survival |
| 25 | 16 | 14 | Cellular Assembly and Organization, Cell cycle, Reproductive System Development and function |

**Supplementary Figure S7. 1. Network diagrams of ingenuity pathway analysis**. Twenty-five clusters were identified including their enrichment score (ES). Each network in a box is displayed according to genes central to the pathway. The top 5 networks (Figure 7.2, 7.3, 7.4, 7.5, and 7.6) for the IL2RG KO pig-derived thymus by IPA analysis. The genes (up-regulated genes indicate red color, but down-regulated genes indicate green color) are networked based on the evidence in the IPA. For further details, see the Materials and Methods section. Network 7.2, 7.3, 7.4, 7.5, and 7.6 are mainly related with immune-related diseases and function.

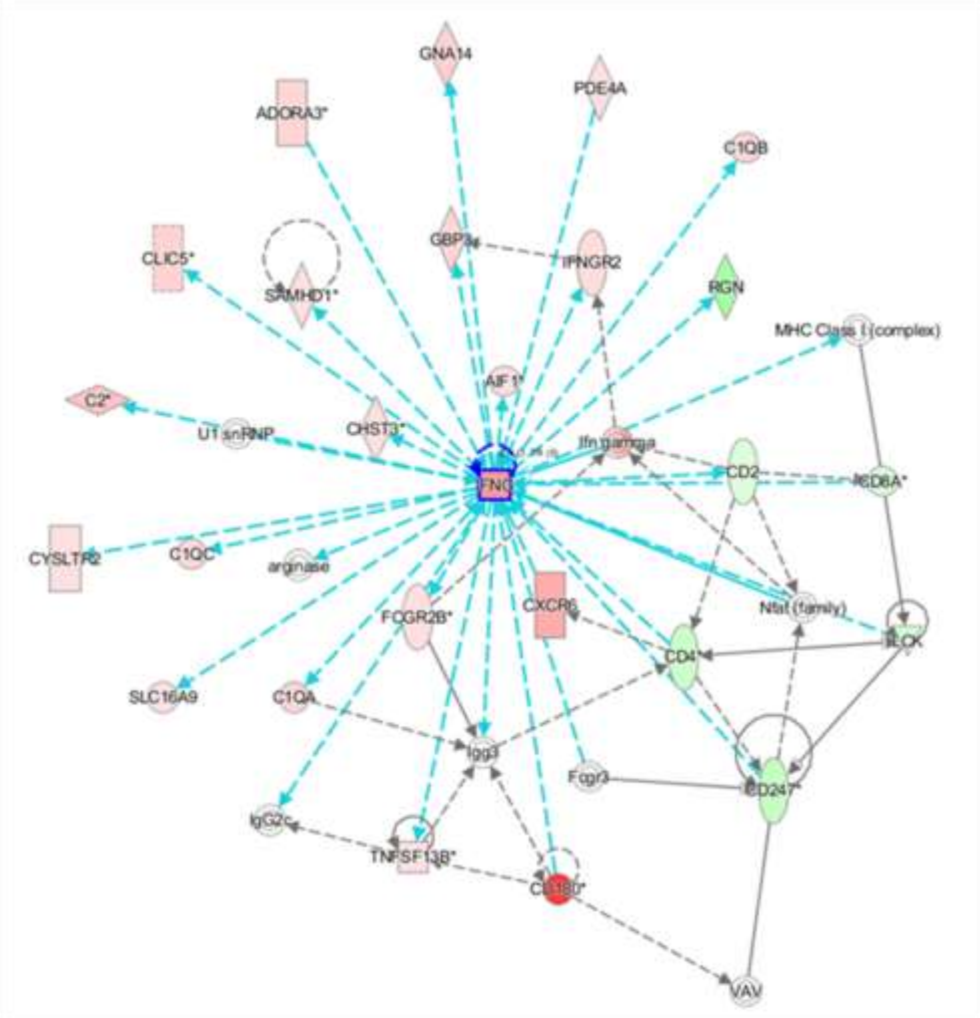


**Supplementary Figure S7.2. Network 4 shows immune-related disease such as immunological disease and inflammatory disease.** The core gene is IFNG and most of related genes are slightly up-regulated than WT. Abbreviations: Guanine nucleotide binding protein Alpha 14 (GNA14), Adenosine A3 receptor (ADORA3), Phosphodiesterase 4A CAMP-Specific (PDE4A), Complement component 1 Q subcomponent B Chain (C1QB), Guanylate binding protein 3 (GBP3), Interferon gamma receptor2 (IFNGR2), RGN, Chloride intracellular channel 5 (CLIC5), SAM domain and HD domain 1 (SAMHD1), Allograft inflammatory factor 1 (AIF1), Carbohydrate sulfotransferase 3 (CHST3), C2, U1SNRNP, Interferon gamma (IFNG), Ifn gamma, MHC class I (complex), Cysteinyl leukotriene receptor 2 (CYSLTR2), Complement component 1 C chain (C1QC), arginase, Fc fragment of

IgG (FCGR2B), Solute carrier family 16 member 9 (SLC16A9), C1QA, Chemokine receptor 6 (CXCR6), Cluster of differentiation 4 (CD4), Cluster of differentiation 2 (CD2), Cluster of differentiation 8a (CD8a), Nuclear factor of activated T-cells (Nfat), Lymphocyte-specific protein tyrosine kinase (LCK), Cluster of differentiation 247 (CD247), Vav 1 oncogene (VAV), Igg3, Fc gamma receptor 3 (Fcgr3), IgG2c, Tumor necrosis factor superfamily member 13b (TNFSF13B), Cluster of differentiation 180 (CD180).

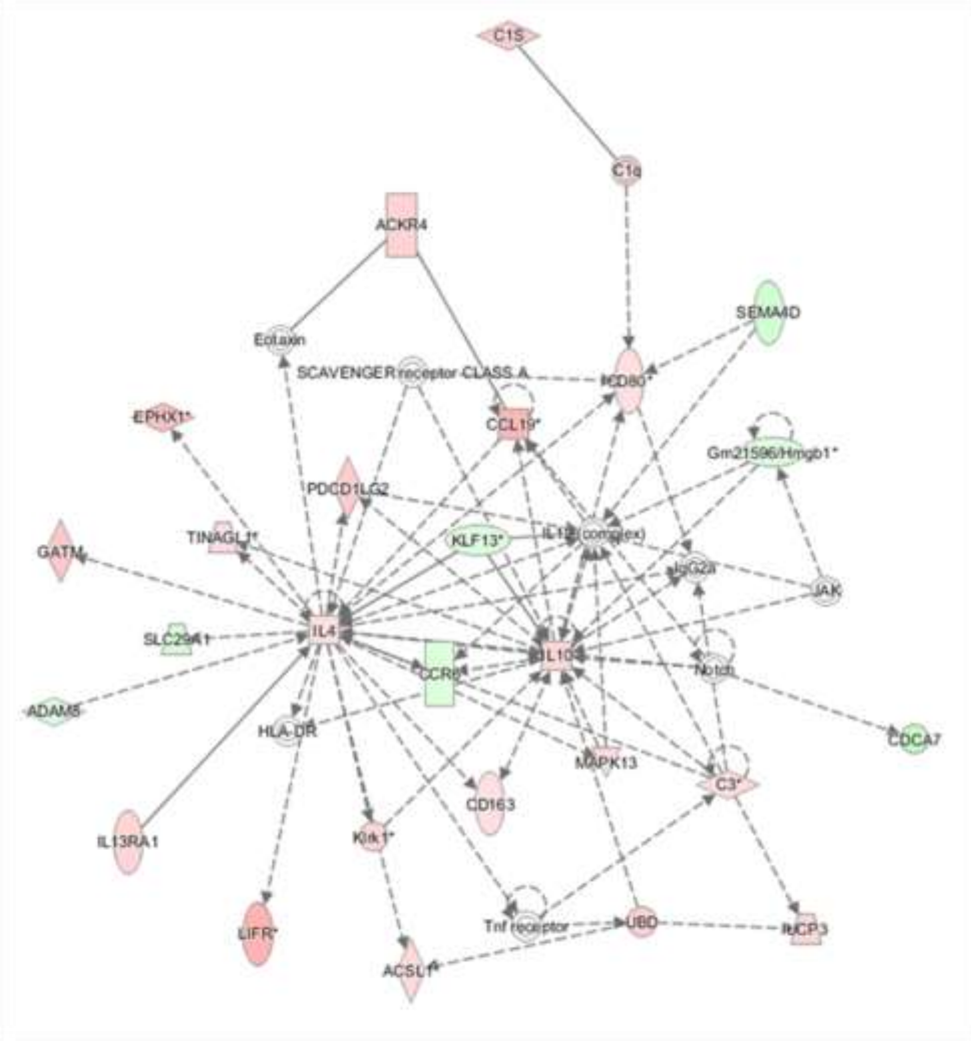


**Supplementary Figure S7.3. Network 5 shows hematological system development and function and immune cell trafficking.** Core genes are IL4 and IL10. Abbreviations: C1S, Atypical chemokine receptor 4 (ACKR4), Semaphorin-4D (SEMA4), Cluster of differentiation 80 (CD80), Chemokine ligand 19 (CCL19), Gm21596/Hmgb1, Eotaxin, Epoxide hydrolase 1 (EPHX1), Programmed cell death 1 ligand 2 (PDCD1LG2), Kruppel-like factor 13 (KLF13), IgG2a, Janus kinase (JAK), Notch, Cell Division cycle associated 7 (CDCA7), Interleukin-10 (IL-10), Mitogen-activated protein kinase 13 (MAPK 13), Complement component 3 (C3), Uncoupling protein 3 ubiquitin D (UBD), Cluster of differentiation 163 (CD163), Chemokine receptor 6 (CCR6), Interleukin 4 (Il4), HLA-DR, KLRK1, Tnl receptor, acyl-CoA synthetase long-chain family member 1 (ACSL1),

Tubulointerstitial nephritis antigen-like 1 (Tinagl1), Solute carrier family 29 member 1 (Slc29a1), Glycine amidinotransferase (GATM), ADAM metallopeptidase domain 8 (ADAM8), Interleukin 13 receptor subunit alpha 1 (IL13RA1), Leukemia inhibitory factor receptor alpha (LIFR).

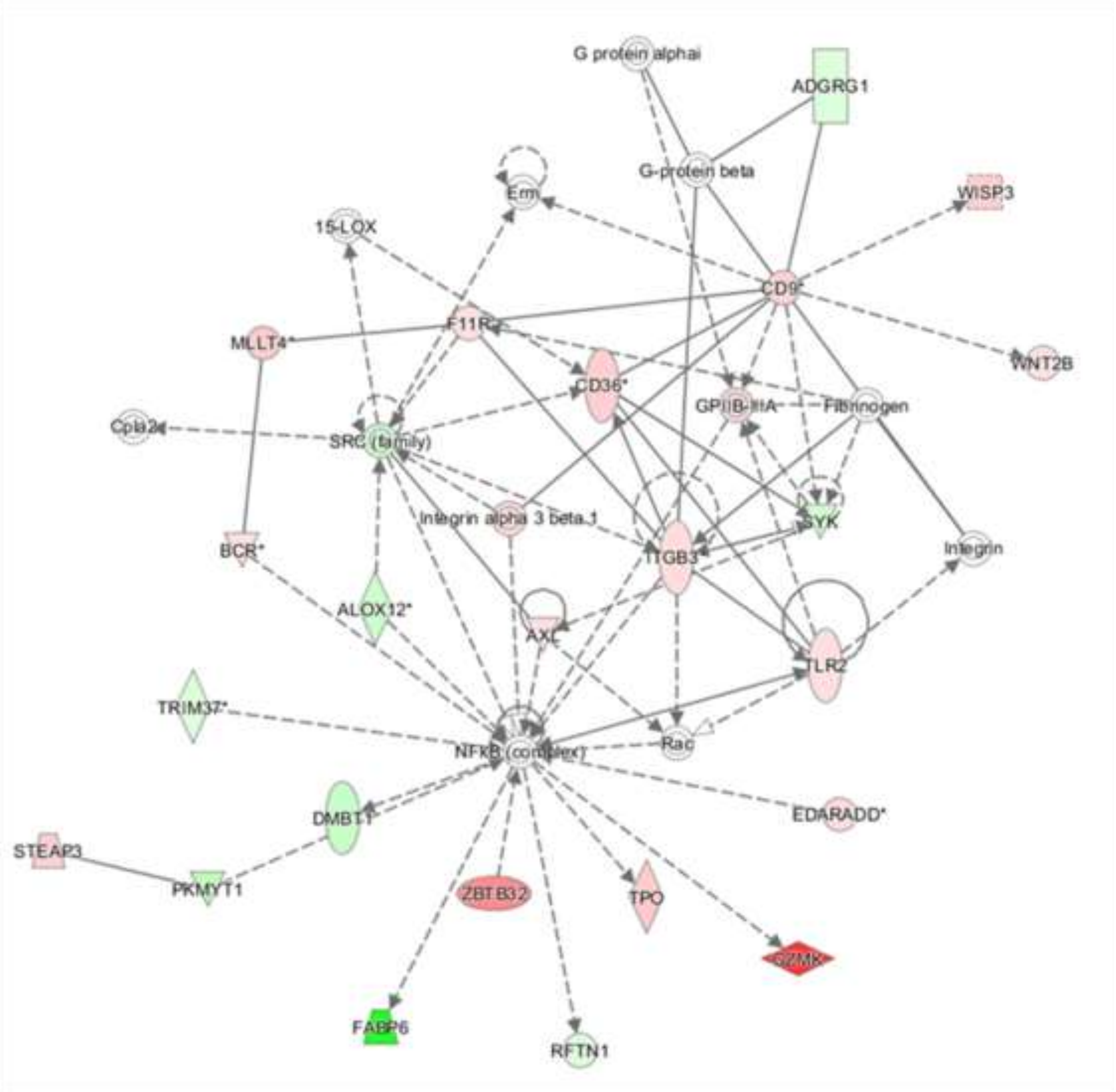


**Supplementary Figure S7.4. Network 9 shows Cell-to-cell signaling and interaction, Cellular function and maintenance, Hematological system development and function**. Core gene is NFkB but it was not detected in microarray data. Abbreviations: G protein alpha i subunit (Galphai), Adhesion G protein-coupled receptor G1 (ADGRG1), WNT1 inducible signaling pathway protein 3 (WISP3), Wingless-type MMTV integration site family member 2B (Wnt2b), Cluster of differentiation 9 (CD9), Fibrinogen C domain-containing protein 1-like (Fibrinogen), Guanine nucleotide binding proteins (G Protein beta), Earmuff (Erm), F11 receptor (F11R), Cluster of differentiation 36 (CD36), GPIIB IIIA, Spleen tyrosine kinase (SYK), Toll-like receptor 2 (TLR2), Integrin subunit beta 3 (ITGB3), RAC, AXL receptor

tyrosine kinase (AXL), Integrin alpha 3 beta1, 15LOX, Myeloid/lymphoid or mixed-lineage leukemia (MLLT4), SRC (family), Nuclear factor kappa B (Nfkb), Arachidonate 12-lipoxygenase (ALOX12), Breakpoint cluster region (BCR), Cytosolic phospholipases A2 (cPLA2), Tripartite motif containing 37 (TRIM37), Deleted in malignant brain tumors 1 (DMBT1), Protein kinase, membrane associated tyrosine/threonine 1 (PKMYT1), STEAP Family Member 3 (STEAP3), Zinc finger and BTB domain containing 32 (ZBTB32), Thyroid peroxidase (TPO), EDAR (ectodysplasin-A receptor)-associated death domain (EDARADD), granzyme K (GZMK), RFN1, Fatty acid binding protein 6 (FABP6).

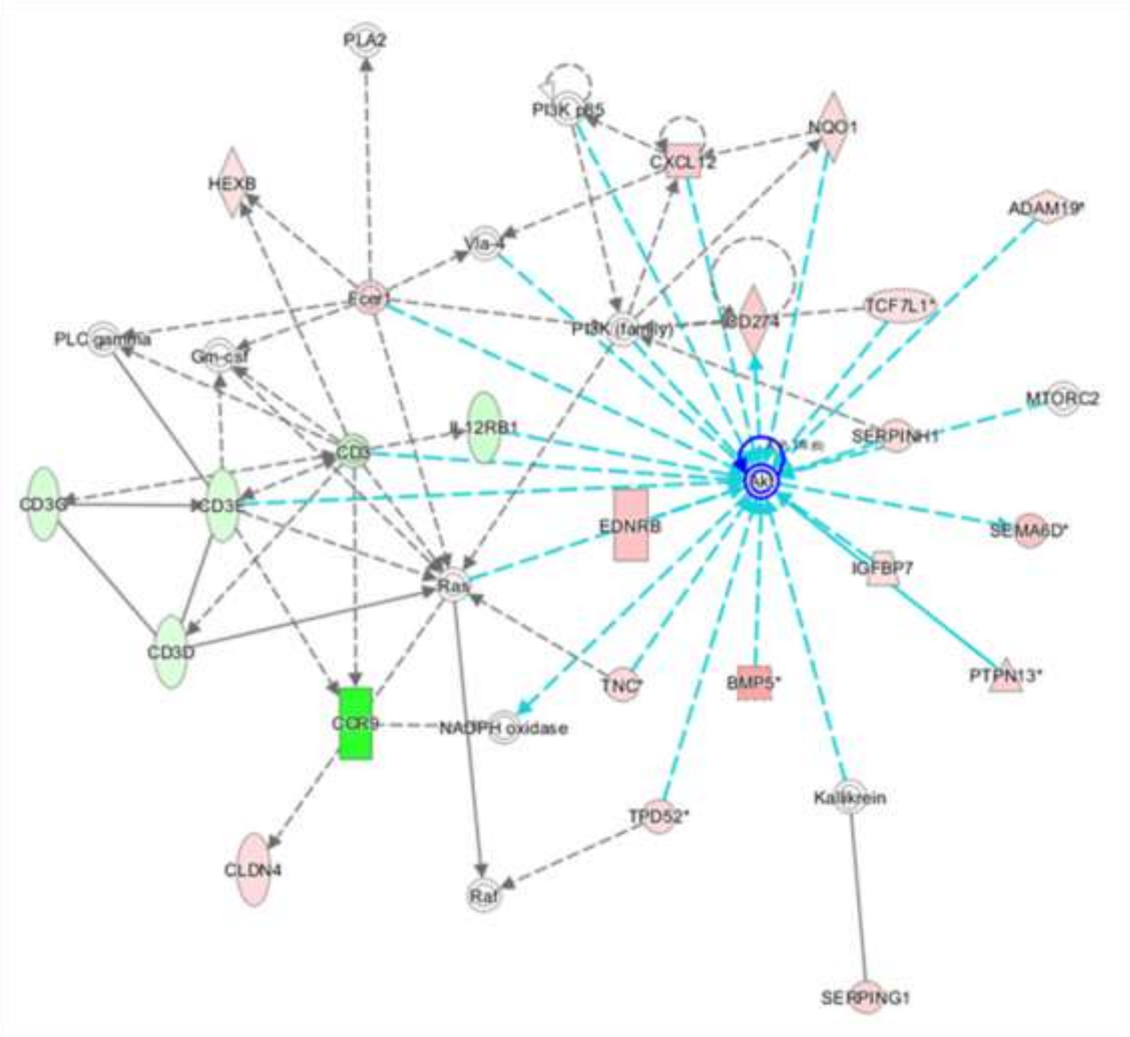


**Supplementary Figure S7.5. Network 14 shows hematological disease and immunological disease.** Core gene is AKT, it was not detected in microarray data. Abbreviations: phospholipase A2 (PLA2), phosphatidylinositol 3-kinase (PI3K) p85 ,chemokine (C-X-C motif) ligand 12 (CXCL12), NAD(P)H dehydrogenase, quinone 1(NQO1), ADAM metallopeptidase domain 19 (ADAM19), transcription factor 7 like 1 (TCF7L1), Cluster of Differentiation 274 (CD274), serpin peptidase inhibitor, clade H (heat shock protein 47), member 1 (SERPINH1), MTORC2, Sema domain 6D (SEMA6D), Insulin like growth factor binding protein (IGFBP7), Protein tyrosine phosphatase, non-receptor type 13 (PTPN13), Bone morphogenetic protein 5 (BMP5), Kallikrein (KLK), Serpin peptidase inhibitor, clade G member 1 (SERPING1), Tumor protein D52 (TPD52), Tenascin C (TNC),

Endothelin receptor type B (EDNRB), Ras, NADPH oxidase, Raf, interleukin 12 receptor subunit beta 1 (IL12RB1), Via4, Phosphatidylinositol 3-kinase (PI3K), Fcer1, Hexosaminidase B (HEXB), Phosphoinositide-specific phospholipase C (PLC), Gm-csl, Cluster of Differentiation 3 (CD3), Cluster of Differentiation 3e (CD3E), Cluster of Differentiation 3g (CD3G), Cluster of Differentiation 3d (CD3D), CDR9, Chemokine (C-C Motif) Receptor 9 (CCR9), Claudin 4 (CLDN4).

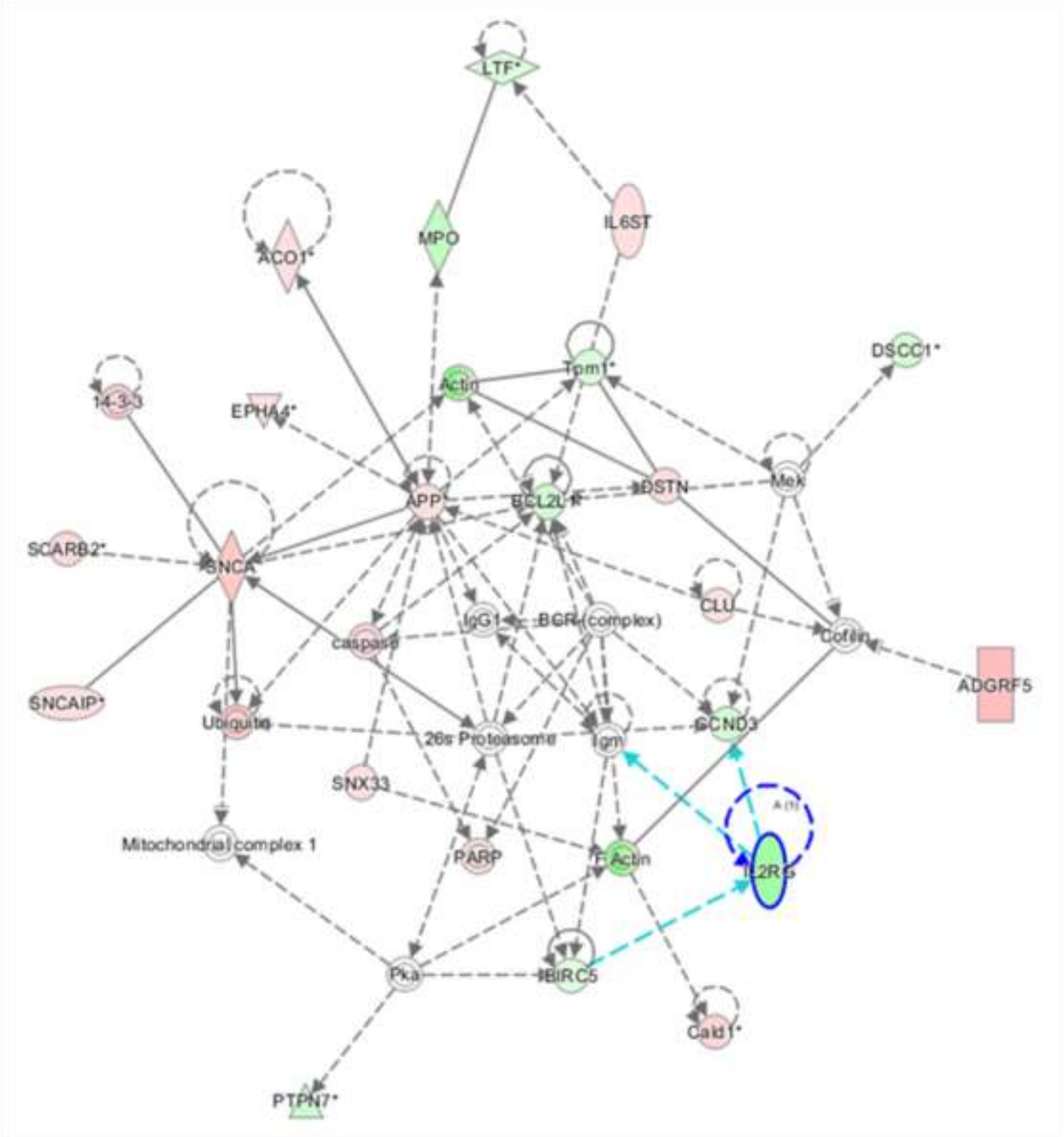


**Supplementary Figure S7.6. Network 15 shows immunological disease and inflammatory disease.** Abbreviations: Lactotransferrin (LTF), Interleukin 6 signal transducer (IL6ST), Myeloperoxidase (MPO), Aconitase 1 (ACO1), DNA replication and sister chromatid cohesion 1 (DSCC1), Tropomyosin 1 (Tpm1), Actin, BCL2-like 1 (BCL2L1), Destrin (DSTN), MAP kinase-ERK kinase (Mek), ClSusterin (CLU), Cofilin, Adhesion G protein-coupled receptor F5 (ADGRF5), GCND3, Interleukin 2 receptor gamma chain (IL2RG), Breakpoint cluster region (BCR), Lgm, F actin, Baculoviral IAP repeat containing 5 (BIRC5), Protein lysine acetyltransferase (Pka), Protein tyrosine phosphatase,

non-receptor type 7 (PTPN7), Sorting nexin 33 (SNX33), Caspase, Ubiquitin, synuclein alpha (SNCA), Eph receptor A4 (EPHA4), 14-3-3, Scavenger receptor class B member 2 (SCARB2), Synuclein alpha interacting protein (SNCAIP), 26s proteasome, IgG1, Ayloid beta precursor protein (APP), Mitochondria complex 1, Poly- polymerase (PARP).

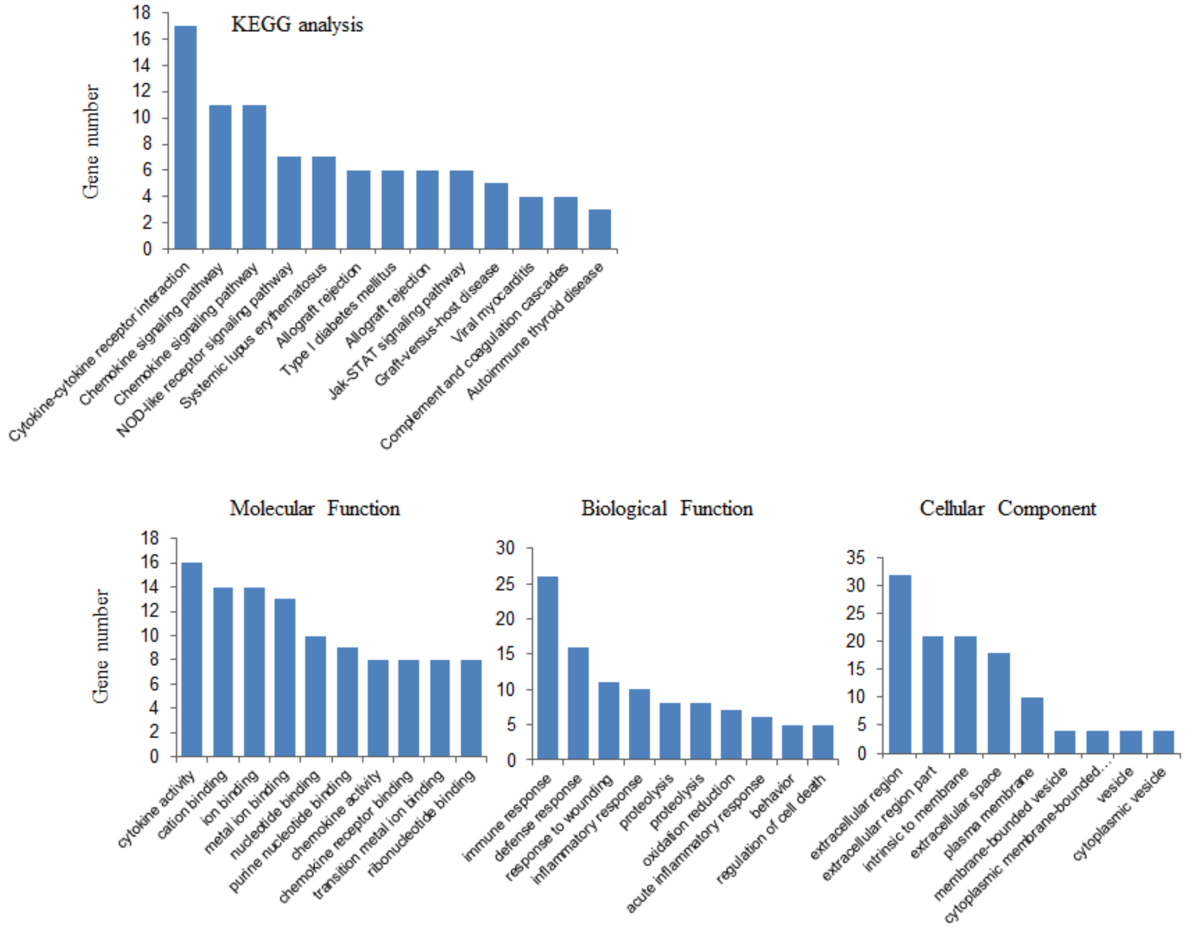


**Supplementary Figure S8. KEGG pathway enrichment analysis, GO biology process,** molecular function, and cellular function analysis in WT and mIL2RG$^{+/\Delta 69\text{-}368}$ KO pig spleen. Using DAVID program, up- and down- regulated gene expressions were divided into 3 subgroups such as Biological process, Cellular component, and Molecular function. Each subgroup shows top 10 of gene function group.

A

| ID network | # molecules | Score | Disease and function |
|---|---|---|---|
| 1 | 22 | 36 | Cell-to-cell signaling and interaction, cellular movement, immune cell t rafficking |
| 2 | 21 | 33 | Infectious disease, connective tissue disorders, inflammatory disease |
| 3 | 20 | 31 | Hematological system development and function, tissue morphology, ce llular function and maintenance |
| 4 | 19 | 29 | Infectious disease, cell morphology, cell-to-cell signaling and interactio n |
| 5 | 16 | 23 | Cellular movement, Hematological system development and function, i mmune cell trafficking |
| 6 | 13 | 17 | Dermatological diseases and conditions, reproductive system developm ent and function, immunological disease |
| 7 | 12 | 15 | Cellular development, connective tissue development and function, skel etal and muscular system development and function |
| 8 | 11 | 14 | Cell morphology, cellular compromise, cellular function and maintenan ce |
| 9 | 11 | 14 | Embryonic development, hair and skin developmetn and function, orga n development |
| 10 | 8 | 9 | Drug metabolism, molecular transport, small molecule biochemistry |
| 11 | 1 | 2 | Cancer, cardiovascular disease, cell cycle |
| 12 | 1 | 2 | Development disorder, ophthalmic diseas, organismal injury and abnor malities |
| 13 | 1 | 2 | RNA post-transcriptional modification, DNA replication, recombinatio n, and repair, cell death and survival |

B

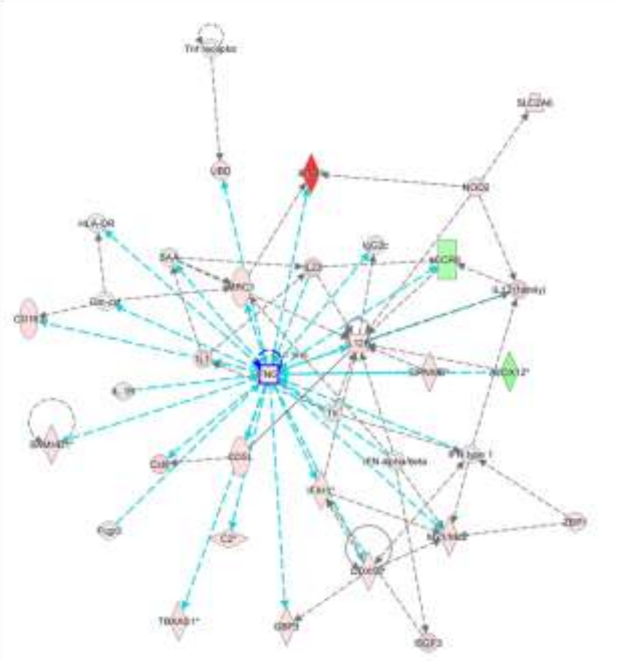

**Supplementary Figure S9. Network analysis by IPA program in spleen**. (a) Summary of each network. Network 1-6 have immune-related diseases and function. (b) In Network 4, core gene is IFNG and most of related genes are slightly up-regulated than WT. Abbreviations: Tnf receptor, Ubiquitin D (UBD), Major histocompatibility complex (HLA-DR), Cluster of differentiation 163 (CD163), Granulocyte-macrophage colony-stimulating factor (Gm-csf), Mannose receptor C type 1 (MRC1), Immunoresponsive gene 1 (IRG1), Interleukin 23 (IL23), IgG2c, Solute carrier family 2 member 6 (SLC2A6), Nucleotide-binding oligomerization domain-containing protein 2 (NOD2), Chemokine receptor 6 (CCR6), Interleukin 12 (IL12) family, Interleukin 12 beta (IL12B), Interleukin 1 (IL1), Interleukin 1 receptor (IL1R), SAM domain and HD domain-containing protein 1 (SAMHD1), Chemokine (C-C Motif) ligand 8 (Ccl8), CD5 Molecule-Like (CD5L), Interferon gamma (IFNG), Toll-like receptors (Tlr), Glycoprotein nmb (GPNMb), Arachidonate 12-lipoxygenase (ALOX12), Fcg receptor III (Fcgr3), Complement component 2 (C2), Thromboxane A synthase 1 (TBXAS1), Guanylate binding protein 3 (GBP3),

Interferon-induced helicase (IFIH1), DEAD box polypeptide 58 (DDX58), Interferon alpha/beta (IFN alpha/beta), Type I interferons (IFN type1), MX dynamin-Like GTPase 1/2 (Mx1/Mx2), Z-DNA binding protein 1 (ZBP1), IFN-stimulated gene factor 3 (ISGF3).

10.1Proliferation of immune cell

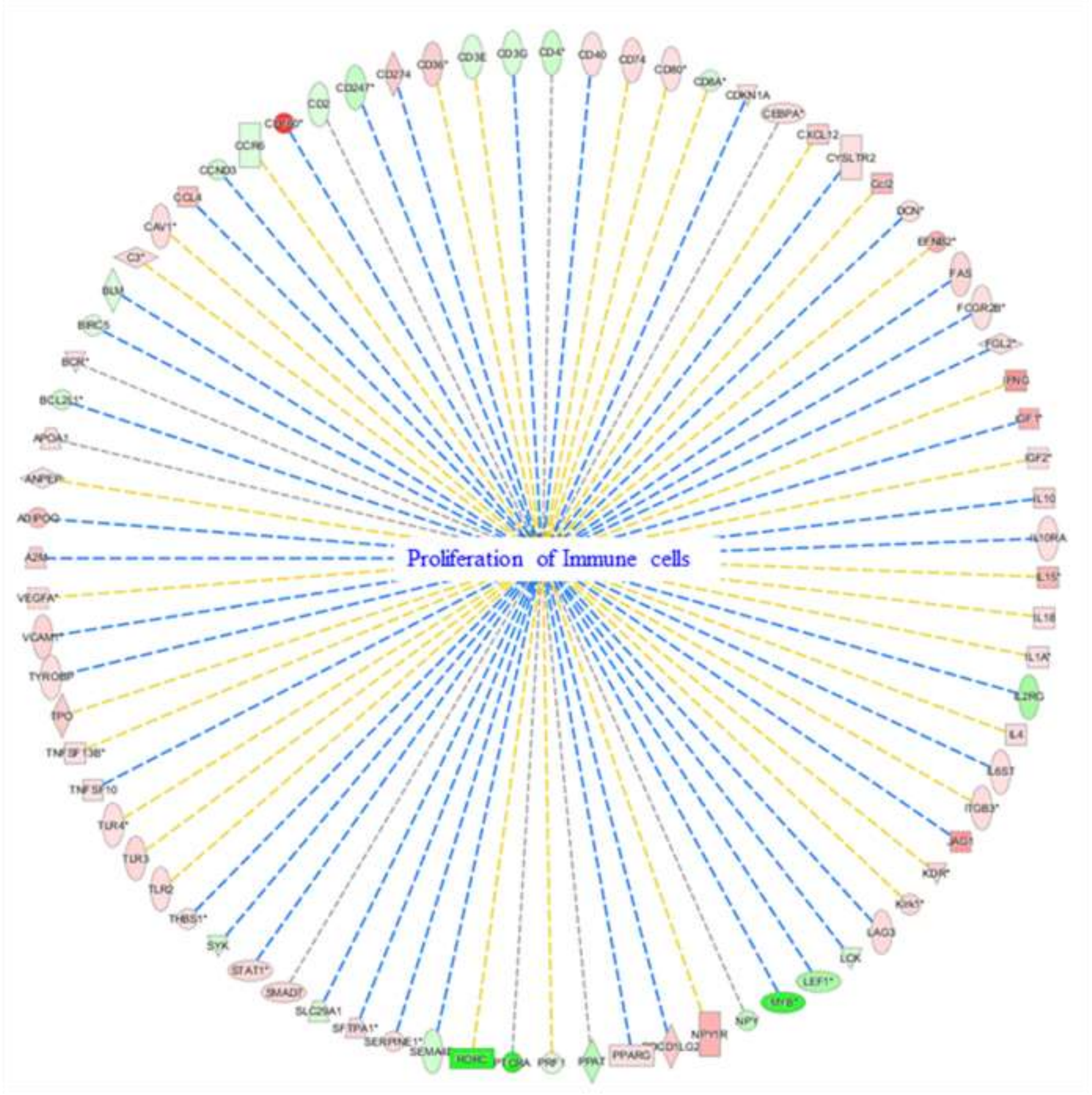


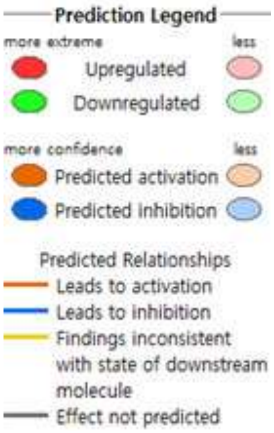

10.2 Quantity of T lymphocytes

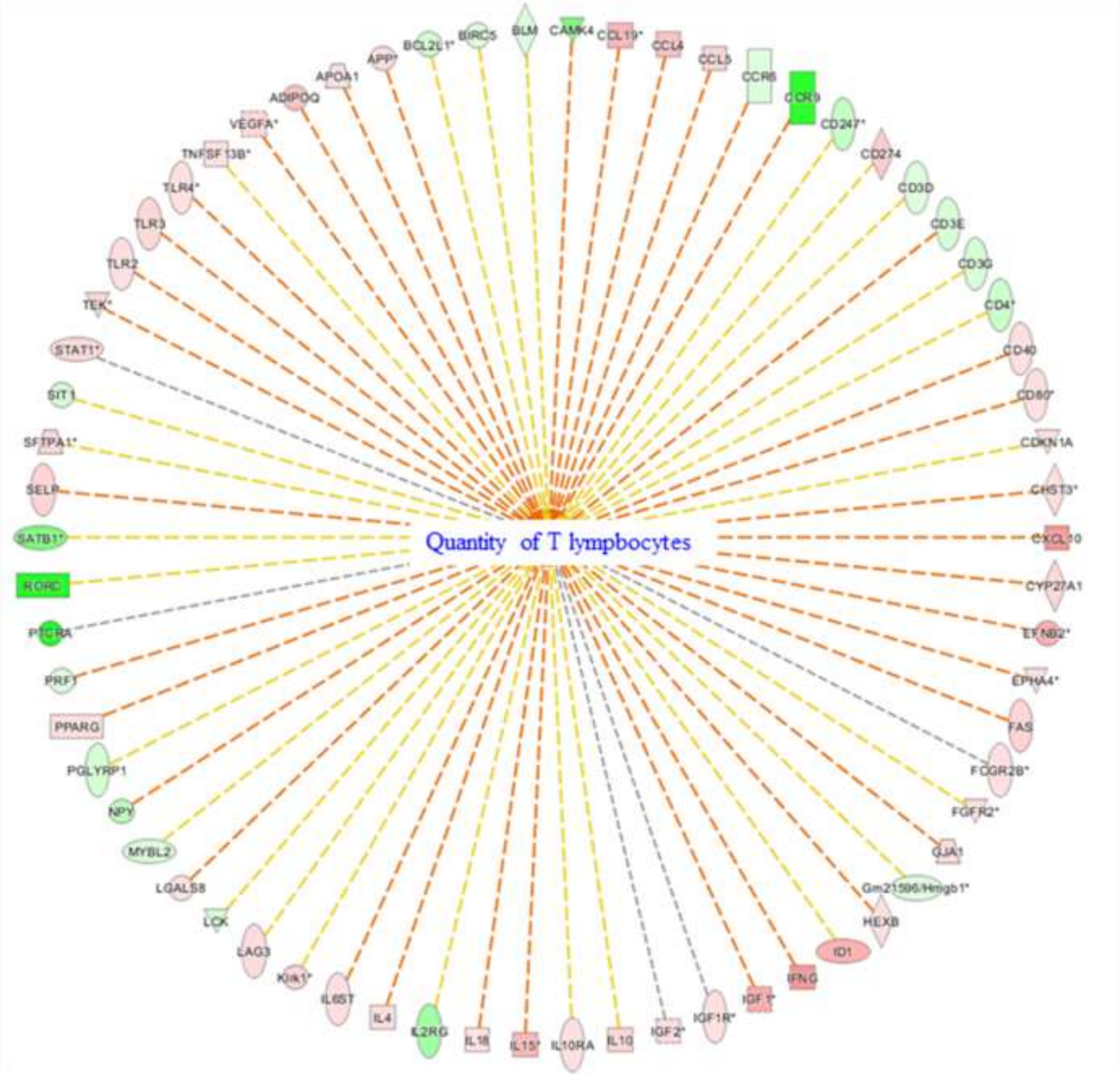

10.3 Inflammation of organ

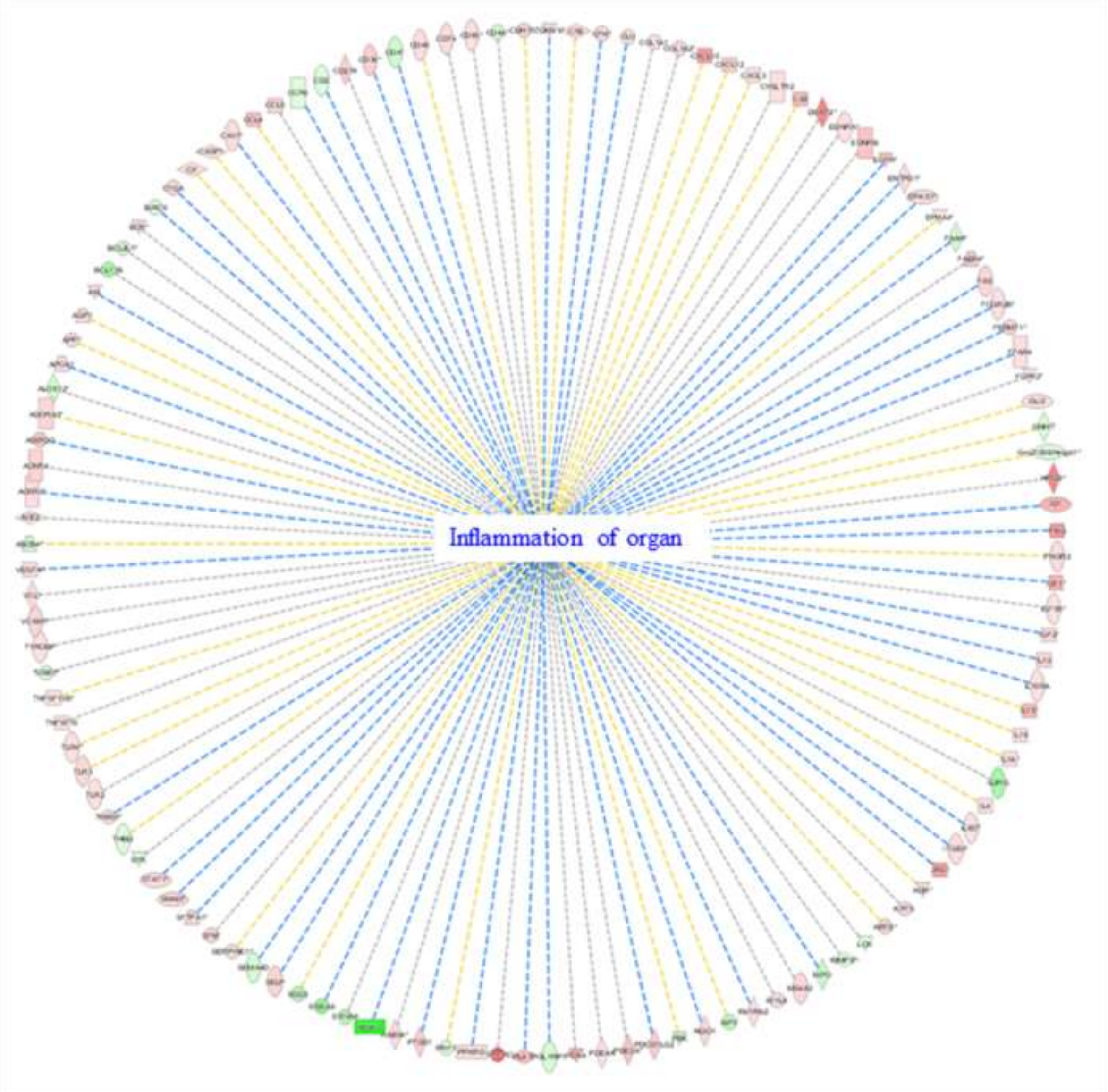

10.4 Infilteration

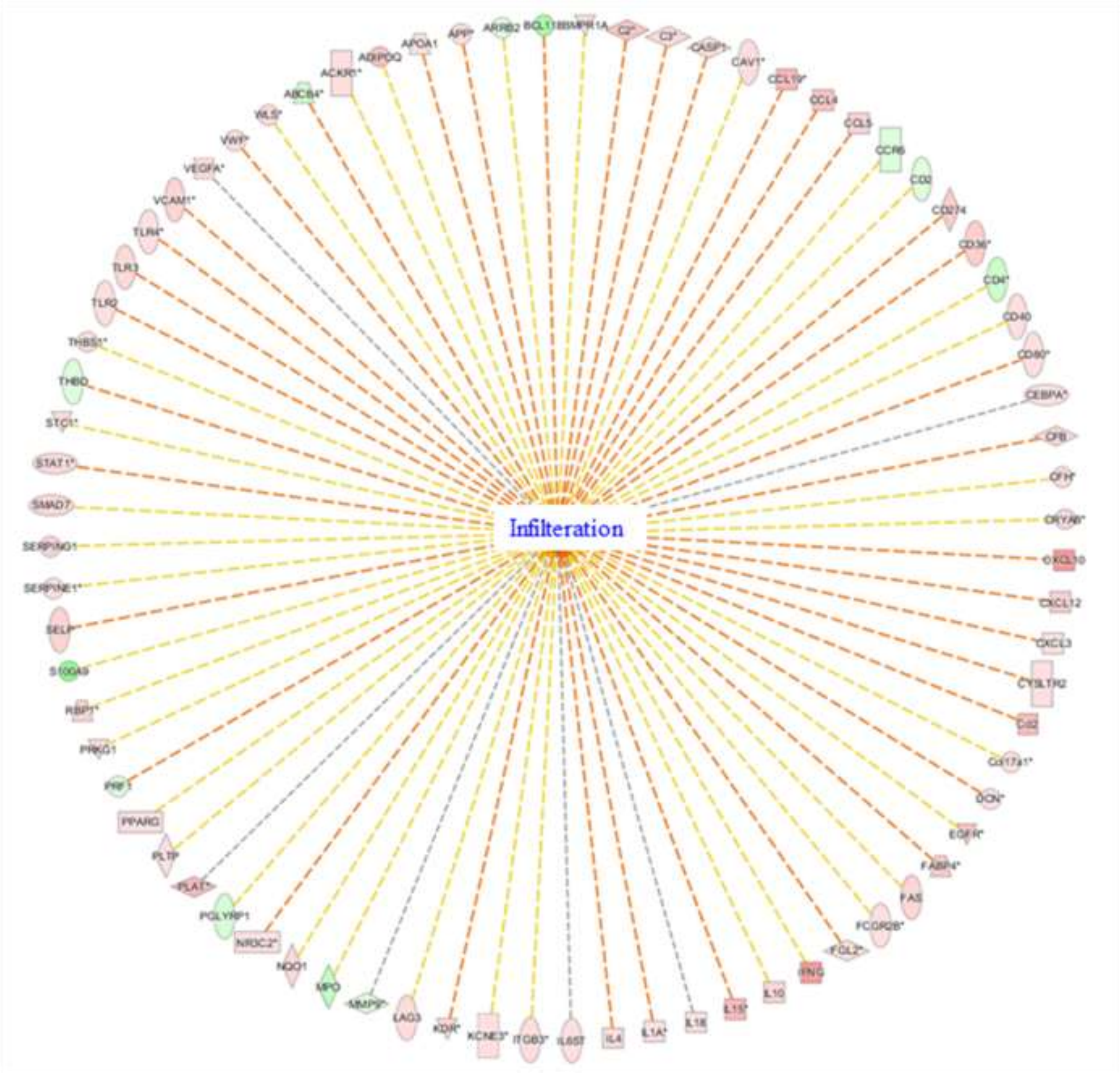


**Supplementary Figure S10. Network 10.1 to 10.4 displays the relationship between proliferation of immune cells, quantity of T lymphocytes, inflammation of organ, infiltration and their target molecules in IL2RG KO pig-derived thymus**. The colors indicate the level of mRNA expression: upregulated genes are represented in red and downregulated genes in green. Most immune-related genes were up-regulated compared to WT.
